# Nitric oxide mediates glial-induced neurodegeneration in Alexander disease

**DOI:** 10.1038/ncomms9966

**Published:** 2015-11-26

**Authors:** Liqun Wang, Tracy L. Hagemann, Hermann Kalwa, Thomas Michel, Albee Messing, Mel B. Feany

**Affiliations:** 1 Department of Pathology, Brigham and Women's Hospital, Harvard Medical School, Boston, Massachusetts 02115, USA; 2 Waisman Center, Department of Comparative Biosciences, and School of Veterinary Medicine, University of Wisconsin-Madison, Madison, Wisconsin 53705, USA; 3 Cardiovascular Division, Department of Medicine, Brigham and Women's Hospital, Harvard Medical School, Boston, Massachusetts 02115, USA

## Abstract

Glia play critical roles in maintaining the structure and function of the nervous system; however, the specific contribution that astroglia make to neurodegeneration in human disease states remains largely undefined. Here we use Alexander disease, a serious degenerative neurological disorder caused by astrocyte dysfunction, to identify glial-derived NO as a signalling molecule triggering astrocyte-mediated neuronal degeneration. We further find that NO acts through cGMP signalling in neurons to promote cell death. Glial cells themselves also degenerate, via the DNA damage response and p53. Our findings thus define a specific mechanism for glial-induced non-cell autonomous neuronal cell death, and identify a potential therapeutic target for reducing cellular toxicity in Alexander disease, and possibly other neurodegenerative disorders with glial dysfunction.

## Abstract (web summary)

Alexander disease is a rare neurological disorder caused by mutations in GFAP, yet it is unclear how glial disruptions lead to neural death. Here, Wang *et al*. identify a mechanism by which glial-derived nitric oxide leads to neuronal degeneration in fly and mouse models of the disease.

## Graphical abstract

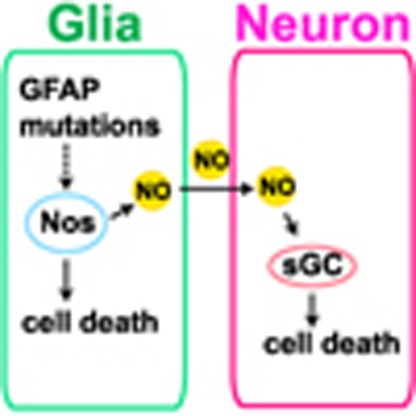

Astrocytes are now known to influence a variety of complex nervous processes including sleep, learning and memory, and breathing^1^,^2^. At the molecular and cell biological levels, astrocytes play a critical role in regulating the permeability of the blood–brain barrier, responding to oxidative stress, modulating synaptic transmission and controlling synaptogenesis and synaptic pruning. Given the plausible role many of these processes play in neuronal dysfunction and death during neurodegeneration, astrocytes seem poised to be critical players in neurodegenerative disorders. Indeed, morphological changes in astrocytes are prominent in all neurological disorders characterized neuropathologically by significant cell loss or tissue damage. However, rigorously defining a role for astrocytes in neurodegenerative disorders has been challenging. First, in most disorders, neuronal pathology is quite prominent, raising the possibility that changes in glial cells are secondary phenomena. Second, it has been challenging to identify the molecular underpinnings of glial-induced neuronal death and test cell-type-specific mechanisms *in vivo*.

To address these limitations, we have developed a model of a glial-based neurodegenerative disorder, Alexander disease, in the genetically accessible model organism *Drosophila*. Alexander disease offers a unique opportunity to study glial-mediated neurodegeneration, because the primary abnormality in patients is glial: dominant missense mutations in the gene encoding glial fibrillary acidic protein (GFAP), the intermediate filament of astrocytes^3^. Clinical presentations of this rare, but informative, disorder range from infants with prominent leukodystrophy and seizures to adults with relative preservation of myelin and a movement disorder^4^,^5^. In all cases, the specific neuropathological manifestation of Alexander disease is the formation of eosinophilic, beaded inclusions termed Rosenthal fibres within astrocytes. Given evidence from patients and mouse models^3^,^6^,^7^ for a toxic dominant gain-of-function mechanism related to abnormal aggregation and toxicity of GFAP in Alexander disease, we have modelled the disorder by expressing disease-linked forms of human GFAP in fly glia^8^. Our *Drosophila* model replicates key features of the human disorder, including formation of Rosenthal fibre-like inclusions, seizures and non-cell autonomous neurodegeneration. At the molecular level, protein misfolding, oxidative stress and dysregulated glutamate transport are critical mediators of disease pathogenesis in our fly model, correlating well with results from mice and analysis of human tissue^7^,^9^,^10^,^11^.

An important, perhaps the most important, strength of studying human disease in *Drosophila* is the ability to perform unbiased forward genetic screens, which have the potential to provide hypothesis-independent insights into disease pathogenesis. Accordingly, we have performed genetic screening in our fly model of Alexander disease. Through our screen, we identify nitric oxide (NO) as a critical mediator of non-cell autonomous neurodegeneration. Further, to dissect the cell-type specificity of the effect, we develop a system to manipulate gene expression independently in neurons and glia. Using a newly developed dual-expression system, we demonstrate a key role for NO synthesized and released in glia acting through neuronal NO targets to promote neuronal cell death.

## Results

### NO signalling is upregulated in *Drosophila* Alexander disease

Our *Drosophila* model of Alexander disease is based on expression of human disease-associated mutant GFAP^R79H^ in fly glia using the standard GAL4/UAS bipartite expression system^12^ and the glial-specific driver *repo-GAL4* (refs 8, 13). Our model recapitulates multiple aspects of authentic Alexander disease, including glial toxicity and secondary non-cell autonomous neurodegeneration^8^. To identify genetic modifiers of GFAP toxicity in an unbiased pattern, we performed an unbiased forward genetic screen using a collection of 2,239 transgenic RNA interference (RNAi) lines^14^. To monitor toxicity of GFAP to glia under screening conditions, we used the transgenic caspase reporter developed by Williams *et al*.^15^ By monitoring caspase activation in 10-day-old adult flies expressing GFAP^R79H^ and RNAi targeting individual genes in glial cells, we recovered a number of candidate suppressors and enhancers of GFAP toxicity. One of these candidate suppressors was nitric oxide synthase (Nos).

**Figure 1:**
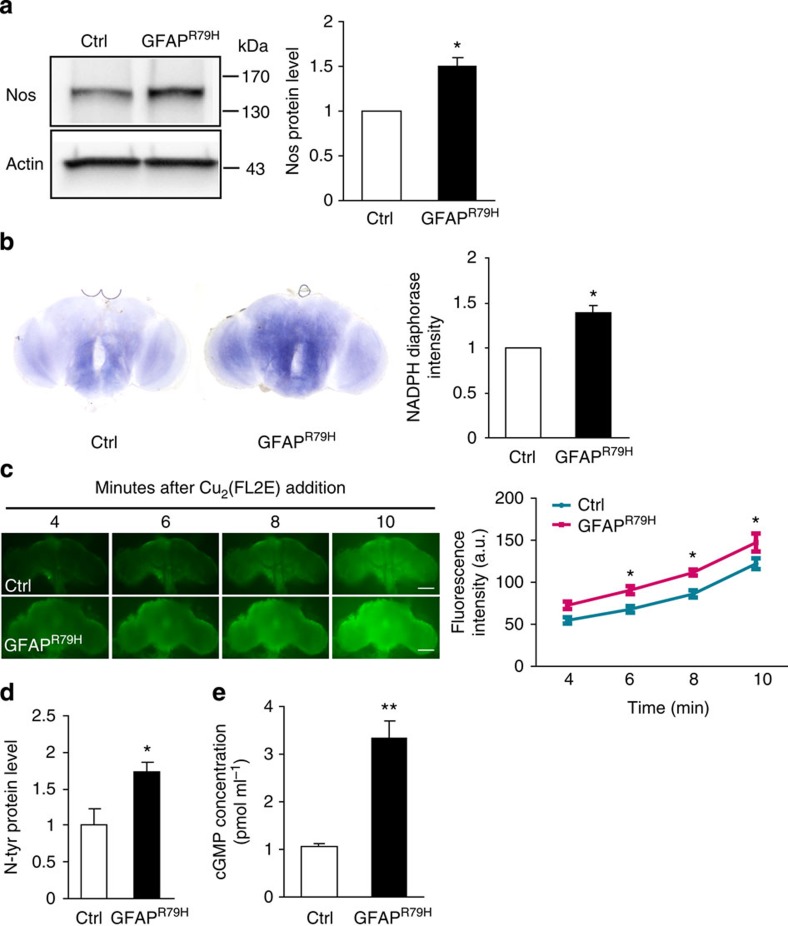
NO signalling is upregulated in GFAP^R79H^ transgenic flies. (**a**) Western blot shows increased expression of Nos in GFAP^R79H^ transgenic flies. The blot was reprobed for actin to illustrate equivalent loading. Quantification of Nos protein levels in control and GFAP^R79H^ transgenic flies is shown in the graph at the right. *P=0.0078, *t*=6.371, df=3; unpaired *t*-test, *n*=4. (**b**) NADPH-diaphorase staining is increased in the brain of GFAP^R79H^ transgenic flies. Quantification is shown in the graph at the right. *P=0.0021, *t*=5.800, df=5; unpaired *t*-test, *n*=6 per genotype. (**c**) Time-lapse imaging reveals increased nitric oxide production *in vivo* in GFAP^R79H^ transgenic flies with a nitric oxide sensor Cu_2_(FL2E). Scale bar, 10 μm. *P<0.05, *t*=2.833 (6 min); 3.173 (8 min); 3.137 (10 min); two-way ANOVA, *n*=7 per genotype. (**d**) Nitrotyrosine (N-tyr)-modified proteins are increased in GFAP^R79H^ flies. *P=0.0273, *t*=2.902, df=6; unpaired *t*-test, *n*=4 per genotype. (**e**) Significantly increased cGMP in GFAP^R79H^ transgenic flies as assayed by the chemiluminescent immunoassay kit. **P<0.0001, *t*=6.109, df=16; unpaired *t*-test, *n*=9 per genotype. Flies were 20 days old in all panels. Control (Ctrl) is *repo-GAL4/+*. GFAP^R79H^ is *repo-GAL4, UAS-GFAP*^*R79H*^*/+*.

Because NO represents a novel, but plausible, glial-derived neuronal cell death signalling molecule in Alexander disease, we explored NO signalling further in our Alexander disease model. We first examined levels of Nos protein (encoded by a single *Nos* gene in flies^16^) in control and GFAP^R79H^ transgenic animals using a rabbit polyclonal antibody to *Drosophila* Nos^17^. A significant increase in Nos in GFAP^R79H^ flies compared with controls was detected by western blot analysis (Fig. 1a). Double-label immunofluorescence using neuronal- and glial-specific markers confirmed the presence of Nos in glial cells (Supplementary Fig. 1a). The specificity of the antiserum was confirmed on both western blot and immunostaining using the *Nos*^*Δ15*^ allele, a deletion mutant of *Nos* removing the entire oxygenase domain^17^ (Supplementary Figs 1a and 2b). To probe Nos activity in our GFAP^R79H^ transgenic flies, we performed nicotinamide adenine dinucleotide phosphate (NADPH)-diaphorase histochemistry^18^. Consistent with Nos protein upregulation, significantly increased NADPH-diaphorase staining was present in GFAP^R79H^ transgenic flies (Fig. 1b). In addition, with a sensitive and specific NO sensor, Cu_2_(FL2E)^19^, we observed significantly increased *in vivo* NO production in the brains of GFAP^R79H^ transgenic flies compared with controls (Fig. 1c). To explore potential mechanisms responsible for upregulation of Nos in our transgenic animals, we evaluated the activity of a well-characterized reporter of STAT expression, 10XSTAT-GFP^20^. We observed upregulation of STAT expression specifically in GFAP^R79H^ transgenic flies (Supplementary Fig. 1b).

We then examined the effect of GFAP^R79H^ expression on cellular pathways downstream of NO production. NO reacts with superoxide (O_2_^−^) to make peroxynitrite (ONOO^−^), a reactive oxidant and cytotoxic agent that can nitrate tyrosine residues of proteins; these modified proteins represent an important indicator of nitrosative stress^21^. Western blot analysis performed with a previously characterized polyclonal anti-nitrotyrosine antibody^22^,^23^,^24^ demonstrated increased levels of nitrotyrosine-containing proteins in GFAP^R79H^ transgenic flies compared with controls (Fig. 1d). Soluble guanylate cyclase (sGC) is an important specific target of NO-mediated nitrosylation^25^. Nitrosylation of sGC activates the enzyme and increases cyclic guanosine monophosphate (cGMP) production. cGMP, in turn, is an important second messenger for a number of downstream targets, including cyclic nucleotide-gated channels (CNGs^25^). Using a sensitive chemiluminescent immunoassay, we found significantly increased levels of cGMP in brains of GFAP^R79H^ flies compared with controls (Fig. 1e).

We also investigated levels of Nos protein, nitrotyrosine-containing proteins and cGMP in transgenic flies that express wild-type human GFAP (GFAP^WT^) at levels equivalent to GFAP^R79H^, and show no evidence of toxicity or inclusion formation (Supplementary Fig. 1f, GFAP^WT^)^8^. We detected no significant difference in Nos protein levels, nitrotyrosine-containing proteins or cGMP levels between GFAP^WT^ transgenic flies and control flies (Supplementary Fig. 1c–e), demonstrating specificity for disease-associated mutant GFAP^R79H^. However, as seen in murine models, increased expression of wild-type human GFAP does result in the formation of Rosenthal fibre-like inclusions (Supplementary Fig. 1f, 2 × GFAP^WT^). We further investigated three additional Alexander disease-associated mutant forms of GFAP, GFAP^R239H^, GFAP^L352P^ and GFAP^A364P^. Each of these mutant forms of GFAP formed Rosenthal fibre-like inclusions in *Drosophila* glia (*repo-GAL4* driver; Supplementary Fig. 1g,h). We also observed inclusion formation when human mutant GFAP was expressed in specific glial subtypes (Supplementary Fig. 1i,j), including astrocyte-like glia (*alrm-GAL4* driver) and ensheathing glia (*mz0709-GAL4* driver)^26^. However, we did not observe significant cellular toxicity with expression in either of these glial subtypes, most likely due to lower levels of transgene expression mediated by these drivers, as demonstrated by reduced β-galactosidase (β-gal) expression mediated by *alrm-GAL4* compared with *repo-GAL4* (Supplementary Fig. 1k).

### Modulation of Nos signalling alters GFAP toxicity *in vivo*

**Figure 2:**
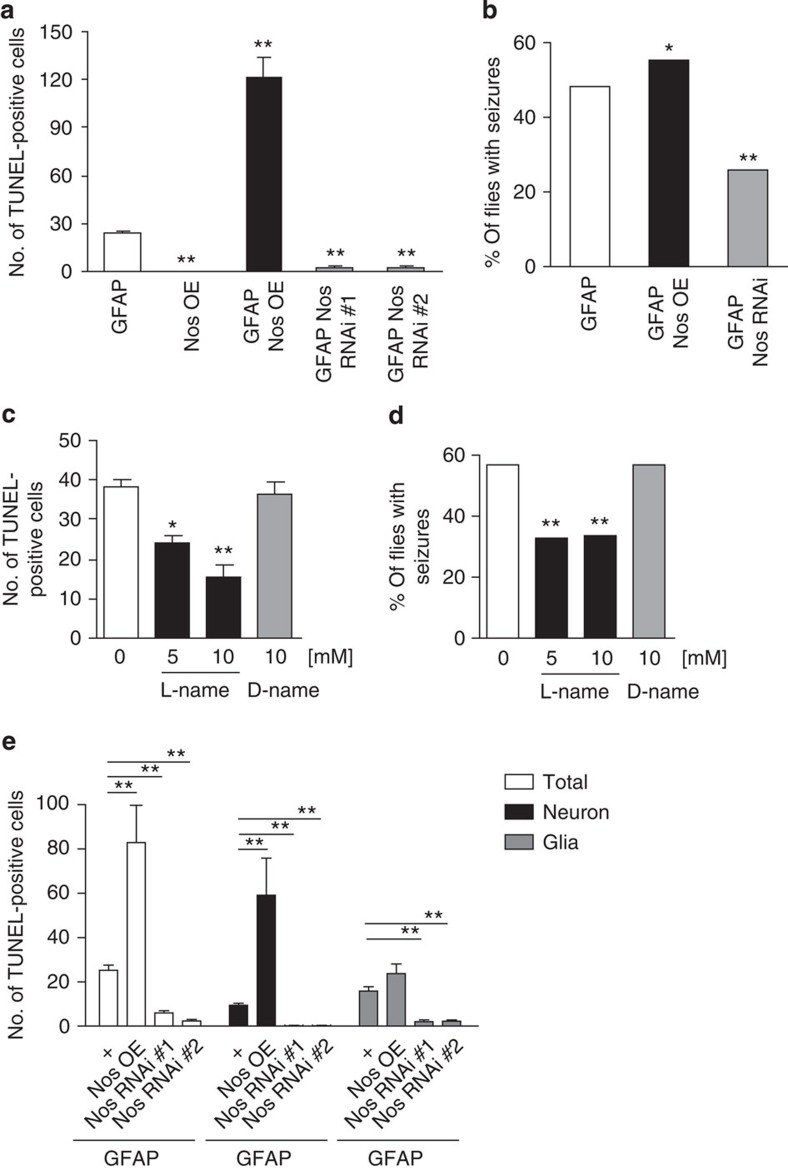
Genetic and pharmacological modulation of Nos activity alters GFAP^R79H^ toxicity. Nos was overexpressed with a *UAS-Nos* transgene (Nos OE) and reduced with *UAS-Nos RNAi* lines (Nos RNAi). GFAP represents *UAS-GFAP*^*R79H*^. (**a**) The number of TUNEL-positive cells is increased in GFAP^R79H^ transgenic flies with overexpression of Nos (Nos OE) and decreased with reduced expression of Nos (Nos RNAi). **P<0.0001, F=72.53, df=29; *n*=6 per genotype. Flies were 20 days old. Genotypes are indicated in the figure label. Driver: *repo-GAL4*. (**b**) Seizure frequency is increased with overexpression of Nos (Nos OE) and decreased with reduced expression of Nos (Nos RNAi). *P=0.0264, *χ*^2^=4.926, df=1; **P<0.0001, *χ*^2^=24.731, df=1; *n*>100 per genotype. Flies were 1 day old. Genotypes are indicated in the figure label. Driver: *repo-GAL4*. (**c**) The Nos inhibitor L-NAME reduces the number of TUNEL-positive cells in GFAP^R79H^ transgenic flies in a dose-dependent manner while D-NAME, the inactive enantiomer, shows no effect. *P<0.01, **P<0.001, F=12.41, df=27; *n*⩾6 per condition. Flies were 15 days old. Genotype: *repo-GAL4, UAS-GFAP*^*R79H*^*/+*. (**d**) Seizure frequency is reduced with L-NAME administration, but not with D-NAME. **P<0.0001, *χ*^2^=24.987, df=1; *χ*^2^=24.083, df=1; *n*>100 per condition. Flies were 3 days old. Genotype: *repo-GAL4, UAS-GFAP*^*R79H*^*/+*. (**e**) Overexpression of Nos (Nos OE) increases, while reduced Nos expression (Nos RNAi) decreases the total number of TUNEL-positive cells (total, left), including both dying neurons (middle) and glia (right) in GFAP^R79H^ transgenic flies. **P<0.0001, F=13.18; 9.627; 17.06; df=23; 23; 23; *n*=6 per genotype. Flies were 20 days old. Genotypes are indicated in the figure label. Driver: *repo-GAL4*. +: *repo-GAL4, UAS-GFAP*^*R79H*^*/+.* Statistical tests: one-way ANOVA with Tukey's multiple comparison test in **a**,**c** and **e**; *χ*^2^-test in **b** and **d**.

To determine whether GFAP toxicity is influenced by Nos activity, we first manipulated the single *Drosophila Nos* gene genetically. Overexpressing Nos in glia using a *UAS-Nos* transgene and the glial driver *repo-GAL4* markedly increased the number of TdT-mediated dUTP nick end labelling (TUNEL)-positive cells, while reducing Nos with either of the two independent transgenic RNAi lines significantly decreased the number of TUNEL-positive cells in the brains of GFAP^R79H^ flies (Fig. 2a,e; Supplementary Fig. 2a). Increasing Nos in the absence of transgenic human GFAP^R79H^ did not produce toxicity (Fig. 2a). Western blot analysis demonstrated marked reduction in Nos levels following RNAi-mediated gene knockdown (Supplementary Fig. 2b). To ensure that modulation of GFAP toxicity was not simply due to alterations in GFAP protein levels, we examined GFAP expression in genetically modified backgrounds and observed equivalent GFAP protein levels (Supplementary Fig. 2c). Consistent with positive TUNEL staining, the nuclei of dying cells were often condensed (43.36±2.06%) and fragmented (32.82±1.64%) in GFAP transgenic flies (Supplementary Fig. 2d). Significant numbers of cells with cleaved caspase (Dcp-1) were present, and activation of *hid* and *th* (also known as *Diap1*), key regulators of apoptosis, was observed using transgenic reporter lines^27^,^28^ (Supplementary Fig. 2e–g). To explore the specificity of our findings, we altered activity of the Nos pathway in flies expressing mutant human ataxin 3, the aggregation-prone polyglutamine expanded protein expressed in patients with spinocerebellar ataxia type 3, in glia^29^. Nos pathway modifiers, including Nos overexpression and knockdown, did not alter the toxicity of mutant ataxin 3 as monitored by vacuolation in the brains of transgenic animals (Supplementary Fig. 2h).

In addition to cell death, our Alexander disease model flies also exhibit seizure activity^8^. We therefore assessed the influence of Nos on seizure frequency in GFAP^R79H^ transgenic flies. As with cell death, increased Nos exacerbated seizure activity, while Nos knockdown decreased seizure frequency (Fig. 2b). Thus, manipulation of Nos levels influences disease-relevant behaviour in our model.

Nos activity can also be controlled pharmacologically. L-NAME is a well-characterized Nos inhibitor with documented activity in *Drosophila*; D-NAME is the inactive enantiomer of L-NAME^30^. To extend our genetic results and assess Nos as a potential therapeutic target, we fed flies orally with either L-NAME or D-NAME. We found that inhibition of Nos activity with L-NAME decreased cell death and seizure frequency, while D-NAME did not alter cell death or the number of seizures in GFAP^R79H^ transgenic flies (Fig. 2c,d; Supplementary Fig. 2i). L-NAME had no effect on the levels of GFAP protein (Supplementary Fig. 2j).

In our fly model of Alexander disease, expression of GFAP^R79H^ specifically in glia triggers non-cell autonomous neurodegeneration^8^, as also occurs in human patients^5^,^31^. Since NO is a diffusible signalling molecule, we hypothesized that NO released from glia triggers neuronal cell death. To begin to test our hypothesis, we examined glial and neuronal cell death in GFAP^R79H^ flies with altered levels of Nos. We and others have previously observed loss of endogenous cell-type-specific markers in apoptotic cells^8^,^32^. We therefore introduced β-gal, expressed using *UAS-lacZ* and *repo-GAL4*, as a transgenic marker and performed double labelling with TUNEL to identify dying glia specifically and distinguish neuronal from glial cell death (Supplementary Fig. 2k). With our cell-type-specific labelling of apoptotic cells, we observed that overexpression of Nos in glia significantly promoted neuronal cell death, consistent with a non-cell autonomous effect of glial NO release (Fig. 2e, middle).

However, we also observed modestly increased glial cell death with Nos overexpression, as well as reduction in both neuronal and glial cell death with Nos knockdown. These data are consistent with effects of Nos on toxicity in both cell types. Thus, to test the specific role of NO in neurons we wanted to block the effects of glial-derived NO in neurons. Given our evidence for increased cGMP in GFAP^R79H^ transgenic flies (Fig. 1e), we decided to focus on the NO target sGC. In *Drosophila*, genes encoding the alpha and beta subunits of sGC, *Gycα99B* and *Gycβ100B*, have been identified and functionally characterized^33^. To independently and simultaneously manipulate gene expression in neurons and glia, we constructed a dual-glial–neuronal expression system by combining the GAL4/UAS system and the Q system^12^,^34^. The Q system is a bipartite expression system, which is conceptually similar to the GAL4/UAS system but instead relies on the regulatory genes from the *Neurospora qa* gene cluster. The GAL4/UAS and Q systems do not interact and can thus be used for independent simultaneous manipulation of gene expression^34^.

**Figure 3:**
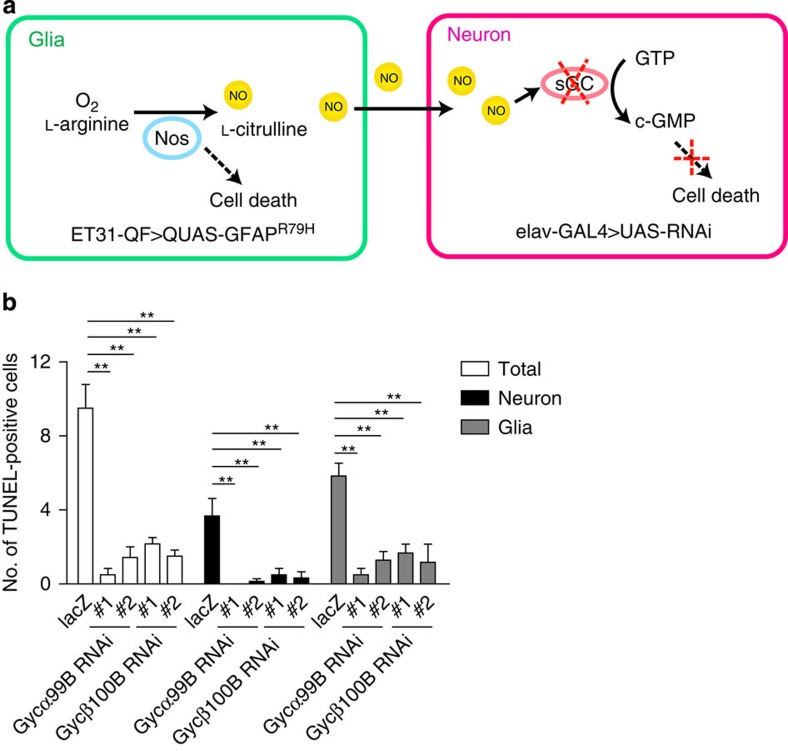
Knockdown of soluble guanylyl cyclase (sGC) in neurons reduces GFAP toxicity. (**a**) Schematic diagram of the dual-transcriptional system. To independently manipulate gene expression in glia and neurons, the GAL4/UAS system and the Q system are used together. GFAP^R79H^ (*QUAS-GFAP*^*R79H*^) is expressed in glia with the Q system glial-specific driver *ET31-QF* (green box) and RNAi lines (UAS-RNAi) targeting sGC are expressed in neurons with the GAL4/UAS system neuronal-specific driver *elav-GAL4* (pink box). Nitric oxide synthase (Nos) produces NO in glia from L-arginine and O_2_. NO diffuses to neighbouring neurons and activates sGC, which converts GTP to cGMP. By expressing RNAi directed to sGC in neurons, NO signalling is blocked. (**b**) Knockdown of two *Drosophila* sGC subunits, Gyc*α*99B and Gycβ100B, with independent RNAi lines, markedly reduces the number of TUNEL-positive cells in GFAP^R79H^ transgenic flies (total, left). Both neuronal (middle) and glial (right) cell death are rescued by reducing expression Gyc*α*99B and Gycβ100B in neurons. **P<0.0001, compared with *UAS-lacZ* control, F=15.32; 11.62; 10.15; df=29; 29, 29. one-way ANOVA with Tukey's multiple comparison test, *n*=6 per genotype. Flies were 20 days old. Genotype: *elav-GAL4; ET31-QF; QUAS-GFAP*^*R79H*^*/UAS-RNAi*. RNAi lines are indicated in the figure label.

Our strategy for independent manipulation of glial and neuronal gene expression is illustrated in Fig. 3a. GFAP^R79H^ (*QUAS-GFAP*^*R79H*^) was expressed in glia using the Q system glial driver *ET31-QF*^34^. These transgenic flies have frequent Rosenthal fibre-like inclusions and increased seizure frequency (Supplementary Fig. 3a–c), recapitulating the phenotypes observed when GFAP^R79H^ is expressed in glia with *repo-GAL4* driver^8^. Transgenic RNAi targeted to *Gycα99B* and *Gyc*β*100B* was then expressed in neurons using the *elav-GAL4* driver. β-Galactosidase was expressed in neurons via *UAS-lacZ* and *elav-GAL4* as a control for expression of an unrelated protein in neurons. Using the dual-expression system, we found that GFAP toxicity was markedly reduced when sGC expression was reduced specifically in neurons using either of the two independent RNAi lines targeting *Gycα99B* or *Gyc*β*100B* (Fig. 3b; Supplementary Fig. 3g, left). Reduction in GFAP toxicity was not due to alterations in GFAP protein levels, as determined by western blot analysis (Supplementary Fig. 3d). We verified significant reductions in messenger RNA for *Gycα99B* and *Gyc*β*100B* with expression of transgenic RNAi targeting these loci using RT–PCR (Supplementary Fig. 3e,f). Taken together, these data support our hypothesis that glial-derived NO activates neuronal sGC to mediate glia-induced non-cell autonomous neuronal cell death. As with manipulation of Nos in glia, sGC knockdown in neurons rescued both neuronal and glial cell death (Fig. 3b; Supplementary Fig. 3g, middle and right). Identification of TUNEL-positive cells as neurons or glia in Fig. 3b was based on morphology, as informed by cell-type-specific labelling experiments (Fig. 2e), given difficulties encountered in introducing an additional transgene into the complex genotypes illustrated in Fig. 3a.

### iNOS signalling is upregulated in Alexander disease mice

**Figure 4:**
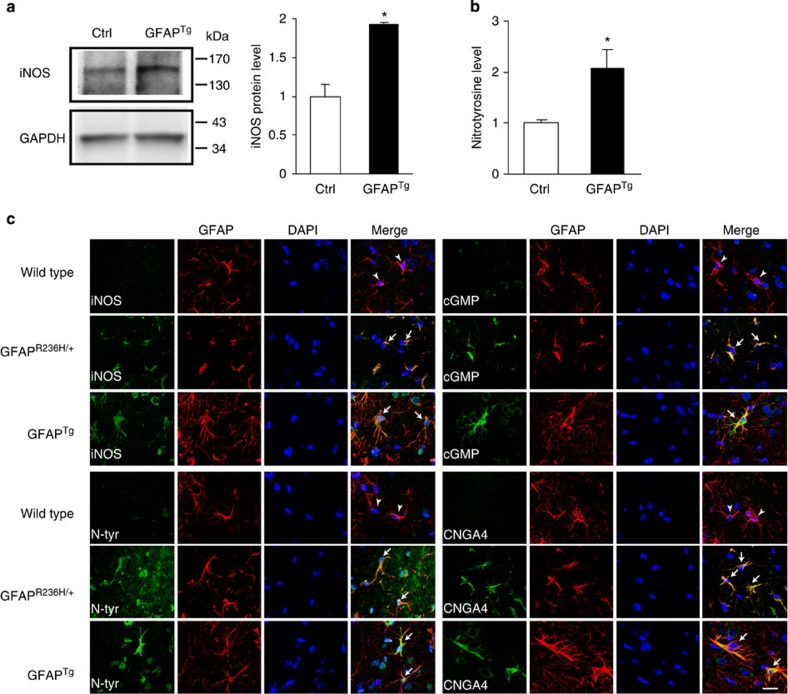
The induced NOS (iNOS) pathway is activated in Alexander disease mouse models. (**a**) Western blot shows increased expression of iNOS in GFAP^Tg^ mice (5 months old). Glyceraldehyde-3-phosphate dehydrogenase (GAPDH) is shown as a loading control. Quantification is shown in the right panel. *P=0.0042, *t*=5.857, df=4; unpaired *t*-test, *n*=3. Ctrl is age-matched wild-type littermates. (**b**) Quantification of nitrotyrosine protein levels reveals an increase in GFAP^Tg^ mice (5 months old). *P=0.0476, *t*=2.825, df=4; unpaired *t*-test, *n*=3. Ctrl is age-matched wild-type littermates. (**c**) Double-label immunofluorescence demonstrates increased expression of iNOS pathway markers: iNOS, nitrotyrosine (N-tyr), cyclic GMP (cGMP) and cyclic nucleotide-gated channel alpha 4 (CNGA4), in astrocytes of 3-month-old GFAP^R236H/+^ and GFAP^Tg^ mice (arrows), but not in age-matched wild-type littermate controls (arrowheads). GFAP was used to identify astrocytes. 4,6-diamidino-2-phenylindole (DAPI) labels nuclei. Scale bar, 20 μm. See Supplementary Figs 8 and 9 for larger images.

To further investigate the role of NO signalling in Alexander disease, we probed the pathway in two well-characterized mouse models of the disorder. Mice expressing either a disease-associated point mutant (GFAP^+/R236H^) or wild-type human GFAP (GFAP^Tg^) develop clinical and pathological features reminiscent of human Alexander disease, with GFAP^Tg^ mice exhibiting more pathology compared with mice heterozygous for the knock-in allele^6^,^7^. Compared with the single *Nos* locus in *Drosophila*, mammals have three distinct *NOS* genes with encoded proteins having different cellular localization, regulation, catalytic properties and inhibitor profiles: neuronal NOS (nNOS or NOS1), inducible NOS (iNOS or NOS2) and endothelial NOS (eNOS or NOS3)^35^. We focused our investigations first on nNOS and iNOS, because in the brain eNOS is mainly expressed in vascular endothelial cells, while nNOS and iNOS are mainly expressed in neurons and astrocytes, respectively, and have been implicated in glial and neuronal apoptosis^35^. Using an nNOS-specific antibody, we were not able to detect differences in nNOS expression between Alexander disease model mice and controls (Supplementary Fig. 4a). In contrast, we observed significant induction of iNOS in Alexander disease model mice. Western blot analysis revealed increased iNOS expression in the cortex of GFAP^Tg^ mice compared with age-matched littermate controls (Fig. 4a). Similarly, double-label immunofluorescence revealed increased iNOS in astrocytes (Fig. 4c; Supplementary Fig. 8), which was validated by a second antibody reagent (Supplementary Fig. 4b).

We also examined downstream components of the pathway and observed a prominent increase in nitrotyrosine-containing proteins by both western blot analysis (Fig. 4b) and immunostaining with a polyclonal anti-nitrotyrosine antibody^22^,^23^,^24^ (Fig. 4c; Supplementary Fig. 8). To confirm and extend prior characterization^22^,^23^,^24^, the anti-nitrotyrosine antibody was validated using nitrated BSA on immunoblot and tissue sections or by reducing nitrotyrosine to aminotyrosine on tissue sections with dithiothreitol in a dose-dependent manner^36^ (Supplementary Fig. 4c). In addition, a second monoclonal anti-nitrotyrosine antibody^37^,^38^ was used for additional confirmation (Supplementary Fig. 4d,e). Similarly, we observed upregulation of cGMP and the CNG channel alpha 4 (CNGA4) in astrocytes (Fig. 4c; Supplementary Fig. 9). Upregulation of each marker was seen in both GFAP^*+/*R236H^ and GFAP^Tg^, with greater increases in GFAP^Tg^ mice compared with GFAP^+/R236H^ animals, consistent with more severe neuropathological changes in the animals overexpressing wild-type GFAP^6^,^7^. iNOS pathway upregulation was seen in multiple regions of the nervous system, including neocortex (Fig. 4c), corpus callosum and hippocampus (Supplementary Fig. 4f). To evaluate the specificity of iNOS induction, we also examined brain tissue from a widely used transgenic mouse model of tauopathy based on expression of human P301L mutant tau. These mice show significant astrogliosis and formation of insoluble tau-containing protein aggregates at 7 months of age^39^. We did not observe significant upregulation of iNOS in astrocytes of tauopathy model mice (Supplementary Fig. 4g).

### Oxidative stress and p53 act downstream of Nos

**Figure 5:**
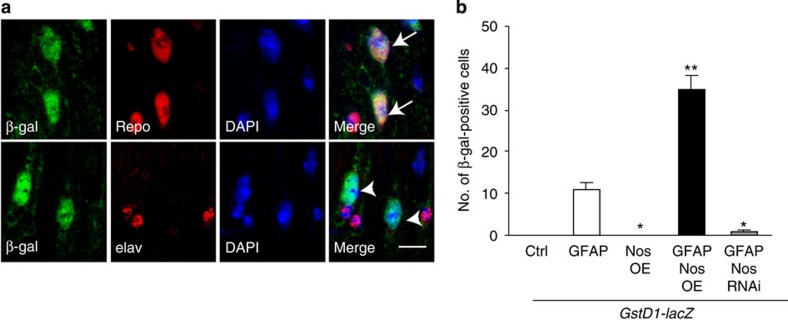
Oxidative stress is regulated by Nos activity in GFAP^R79H^ transgenic flies. (**a**) Double-label immunofluorescence shows activation of the oxidative stress reporter *GstD1-lacZ* in glial cells (top panel, arrows) but not in neurons (bottom panel, arrowheads). Repo marks glial cells; elav marks neuronal cells. 4,6-Diamidino-2-phenylindole (DAPI) labels nuclei. Scale bar, 5 μm. Genotype: *repo-GAL4, UAS-GFAP*^*R79H*^*/+*. Flies were 20 days old. (**b**) Overexpression of Nos in GFAP^R79H^ transgenic flies (GFAP Nos OE) increases the number of β-gal-positive cells, while Nos knockdown (GFAP Nos RNAi) decreases the number of β-gal-positive cells in GFAP^R79H^ transgenic flies. No *GstD-lacZ* activation is detected in Ctrl and Nos OE alone. *P<0.01, **P<0.001 compared with GFAP transgenic flies (GFAP), F=82.35, df=29; one-way ANOVA with Tukey's multiple comparison test, *n*=6 per genotype. Flies were 20 days old. Ctrl is *GstD1-lacZ/+; repo-GAL4/+*. GFAP is *GstD1-lacZ/+; repo-GAL4, UAS-GFAP*^*R79H*^*/+*. Nos OE is *GstD1-lacZ, UAS-Nos/+; repo-GAL4/+*. GFAP Nos OE is *GstD1-lacZ, UAS-Nos/+; repo-GAL4, UAS-GFAP*^*R79H*^*/+*. GFAP Nos RNAi is *GstD1-lacZ/+; repo-GAL4, UAS-GFAP*^*R79H*^*/UAS-Nos RNAi*.

We have previously demonstrated a critical role for oxidative stress in mediating cell death in our *Drosophila* model of Alexander disease^8^. To explore a possible link between Nos and oxidative stress in GFAP^R79H^ transgenic flies, we expressed a sensitive oxidative stress reporter, *GstD1-lacZ*, in which expression of β-gal (*lacZ*) is controlled by the *GstD1* promoter and increases in response to oxidative stress^40^. When we performed double-label immunofluorescence for β-gal and cell-type-specific markers (repo to identify glia and elav to mark neurons), we detected activation of the reporter in glia specifically (Fig. 5a). When we overexpressed Nos together with GFAP^R79H^ in glia using the *repo-GAL4* driver, we further activated the reporter. Conversely, knockdown of Nos decreased reporter activation. Overexpressing Nos in the absence of GFAP^R79H^ did not activate the reporter (Fig. 5b). These data are consistent with a role for oxidative stress downstream of Nos in promoting glial toxicity.

**Figure 6:**
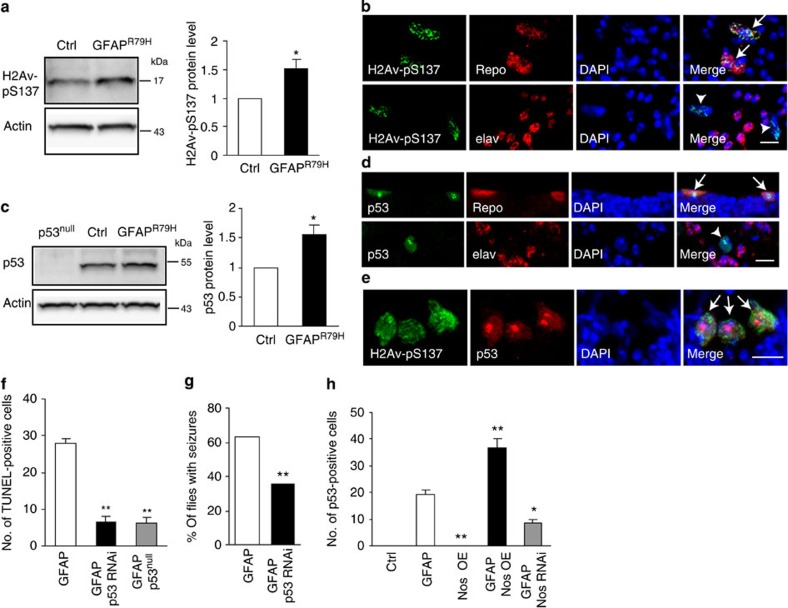
Activation of the DNA damage response and p53 in GFAP transgenic flies. (**a**,**c**) Western blots show significantly increased H2Av-pSer137 and p53 levels in GFAP^R79H^ transgenic flies. The blots were reprobed for actin to illustrate equivalent loading. *P=0.0290, *t*=3.034, df=5, *n*=6 (**a**); *P=0.0356, *t*=3.118, df=4, *n*=5 (**c**); unpaired *t*-test. Ctrl: *repo-GAL4/+*. GFAP^R79H^: *repo-GAL4, UAS-GFAP*^*R79H*^*/+*. (**b**,**d**) H2Av-pS137 and p53 colocalize with the glial cell marker Repo (top panels, arrows) but not with the neuronal cell marker elav (bottom panels, arrowheads) in GFAP^R79H^ transgenic flies. 4,6-Diamidino-2-phenylindole (DAPI) shows nuclei. Scale bar, 5 μm. Genotype: *repo-GAL4, UAS-GFAP*^*R79H*^*/+*. (**e**) Double-label immunofluorescence reveals colocalization of H2Av-pS137 and p53 in GFAP^R79H^ transgenic flies (arrows). DAPI labels nuclei. Scale bar, 5 μm. Genotype: *repo-GAL4, UAS-GFAP*^*R79H*^*/+*. (**f**) Knocking down or removing p53 with transgenic RNAi or a *p53*^*null*^ allele decreases the number of TUNEL-positive cells in GFAP^R79H^ transgenic flies. **P<0.0001, F=74.69, df=18; one-way ANOVA, *n*⩾6 per genotype. Genotypes are indicated in the figure label. Driver: *repo-GAL4*. (**g**) Seizure frequency is decreased with reduced expression of *p53* (p53 RNAi). **P<0.0001, *χ*^2^=45.671, df=1; *χ*^2^-test, *n*>100 per genotype. Genotypes are indicated in the figure label. Driver: *repo-GAL4*. (**h**) Overexpression of Nos (GFAP Nos OE) increases, while reducing Nos (GFAP Nos RNAi) decreases the number of p53-positive cells in GFAP transgenic flies. No p53 accumulation is present in Ctrl and Nos OE alone. *P<0.01, **P<0.0001, F=69.03, df=30; one-way ANOVA, *n*⩾6 per genotype. Genotypes are indicated in the figure label. Driver: *repo-GAL4*. Ctrl: *repo-GAL4/+*. Flies were 1 day old in **g** and 20 days old in other panels.

NO can promote apoptosis through DNA damage and p53 activation^41^. To determine whether DNA damage and p53 activation are mediators of cell death in GFAP^R79H^ flies, we first examined levels of the *Drosophila* DNA damage response marker phosphorylated H2Av (H2Av-pS137) and p53 using western blot analyses. Both markers were significantly increased in GFAP^R79H^ transgenic flies compared with aged-matched control flies (Fig. 6a,c). Immunohistochemical staining also revealed robust activation of H2Av-pS137 and p53 in GFAP^R79H^ transgenic flies (Supplementary Fig. 5a,b). Using double-label immunofluorescence, we determined that both H2Av-pS137 and p53 were increased in glial cells (Fig. 6b,d). Further, H2Av-pS137 and p53 colocalized in the same glial cells (Fig. 6e).

To examine the functional role of p53 in GFAP toxicity, we reduced p53 levels using either a transgenic RNAi line or a *p53* null allele. Both manipulations significantly reduced cell death in GFAP^R79H^ transgenic flies (Fig. 6f). Reducing p53 levels also significantly decreased seizure frequency in GFAP^R79H^ transgenic flies (Fig. 6g), demonstrating a beneficial effect of modulating p53 at the organismal level. The reduction of GFAP toxicity was not due to alterations in GFAP levels, as determined by western blot analysis (Supplementary Fig. 5d). We confirmed decreased (p53 RNAi) or absent (*p53*^*null*^) p53 protein expression using western blotting and a p53-specific antibody (Supplementary Fig. 5e). Reducing p53 levels using transgenic RNAi did not alter the toxicity of mutant ataxin 3, as monitored by vacuole formation (Supplementary Fig. 2d), consistent with specificity of the rescue of GFAP toxicity. To probe the relationship between NO signalling and p53, we counted the number of p53-positive cells in GFAP^R79H^ flies with genetically altered Nos levels. Overexpression of Nos significantly increased the number of p53-positive cells, while knockdown of Nos decreased the number of p53-positive cells (Fig. 6h). These findings together suggest a role for p53-mediated cell death downstream of Nos activation. To further test our model, we determined whether increased levels of p53 can promote death of adult glia in the absence of transgenic GFAP^R79H^. Since overexpression of p53 during development produces organismal lethality, we reduced developmental expression of p53 using the temperature-sensitive GAL4 inhibitor GAL80^ts^ (ref. 42). We then observed TUNEL-positive cell death in flies expressing p53 using the glial *repo-GAL4* driver (Supplementary Fig. 5c). These findings confirm the ability of p53 to promote cell death in adult glia, and taken together with our other results, support a model in which DNA damage and p53 act downstream of Nos to promote glial cell death.

**Figure 7:**
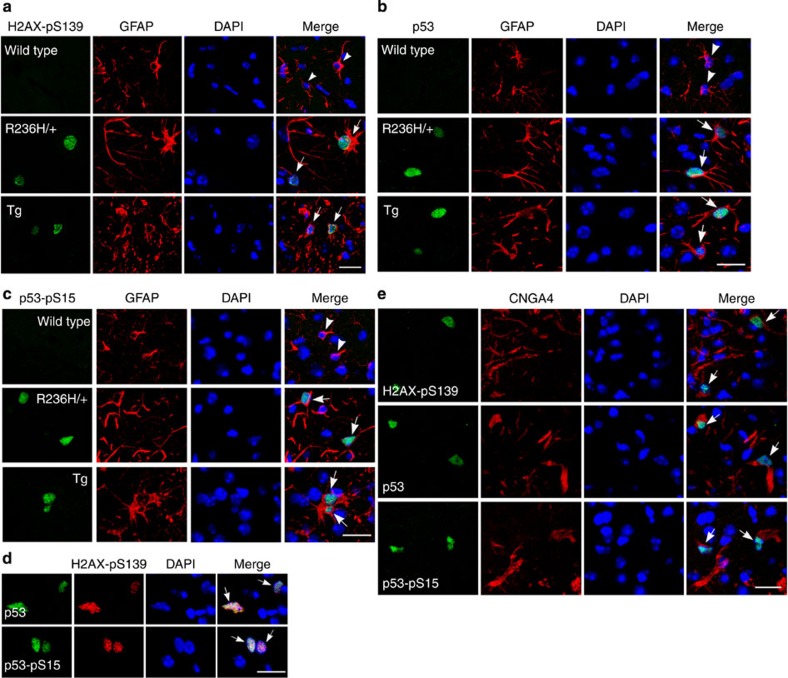
DNA damage response markers are increased in astrocytes of Alexander disease mouse models. (**a**–**c**) DNA damage response markers H2AX-pS139 (**a**), p53 (**b**) and p53-pS15 (**c**) are increased in astrocytes of GFAP^R236H/+^ and GFAP^Tg^ mice (arrows), but not in age-matched wild-type littermate controls (arrowheads). GFAP marks astrocytes. 4,6-Diamidino-2-phenylindole (DAPI) labels nuclei. (**d**) p53 and p53-pS15 colocalize with H2AX-pS139 in GFAP^Tg^ mice (arrows). DAPI labels nuclei. (**e**) H2AX-pS139, p53 and p53-pS15 colocalize with CNGA4 in GFAP^Tg^ mice (arrows). DAPI labels nuclei. Mice were 3 months old in all panels. Scale bar, 20 μm.

We next examined DNA damage and p53 accumulation in mouse models of Alexander disease. Double-label immunofluorescence revealed upregulation of H2AX-pS139, p53 and p53-pS15 in GFAP^+/R236H^ and GFAP^Tg^ mice, but not in age-matched controls (Fig. 7a–c). H2AX-pS139 colocalized with p53 and p53-pS15 in mouse models (Fig. 7d). We also observed colocalization of H2AX-pS139, p53 and p53-pS15 with CNGA4 (Fig. 7e). These data demonstrate activation of the DNA damage response in mouse models of Alexander disease, and are consistent with a role for the pathway downstream of iNOS.

### Increased iNOS level and activated p53 in patient tissue

**Figure 8:**
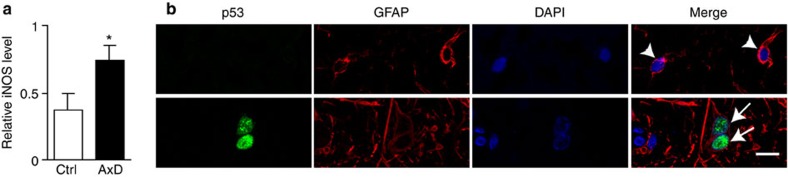
Increased iNOS and p53 in Alexander disease patients. (**a**) Quantification of iNOS protein levels reveals an increase in Alexander disease patients. *P=0.0042, *t*=2.326, df=8; unpaired *t*-test, *n*=5 for both ctrl and AxD. (**b**) Double-label immunofluorescence shows activation of p53 in astrocytes of Alexander disease patient (arrows), but not in control (arrowheads). Images were taken from the grey matter in frontal cortex. GFAP labels astrocytes. 4,6-Diamidino-2-phenylindole (DAPI) marks nuclei. Scale bar, 10 μm.

Having observed upregulation of the iNOS pathway and activation of p53 in *Drosophila*, as well as in mouse models of Alexander disease, we investigated these two pathways in brains from patients with Alexander disease. Western blot analysis of frontal grey matter revealed a statistically significant increase in iNOS expression in Alexander disease patients (Fig. 8a). To evaluate the specificity of our findings, we measured iNOS expression by western blot in frontal grey matter from patients with metachromatic leukodystrophy patients, a childhood neurological disorder with some clinical and pathological features in common with Alexander disease. In contradistinction to our findings in Alexander disease, we observed a decrease in iNOS protein levels in metachromatic leukodystrophy patients (Supplementary Fig. 4h). Consistent with our findings in experimental animal models of Alexander disease, immunohistochemical analysis and double-label immunofluorescence showed a robust and consistent increase in p53 in astrocytes of brain tissue from all Alexander disease patients examined (Fig. 8b).

## Discussion

Although morphological alterations are characteristic of astrocytes (so-called reactive gliosis) in any brain condition with major parenchymal injury, defining the specific role astrocytes play in promoting neuronal pathology has been difficult. Significant neuronal pathology is present in many serious neurological disorders, including neurodegenerative disease, stroke, epilepsy and other brain syndromes. Thus, distinguishing between a primary causal or secondary reactive role for glia in these pathologies has been difficult. To define a primary role of astrocytes in neurodegeneration, we focused here on Alexander disease. Alexander disease is caused by dominant mutations in GFAP, an intermediate filament protein of astrocytes. Therefore, in Alexander disease neurodegeneration is linked directly to astrocyte dysfunction^5^.

Identifying the signalling molecule(s) and pathways that mediate toxic effects of astrocytes on neurons has been a second major challenge in understanding the role astrocytes play in neuronal dysfunction and death^43^. Here we have used a fly model of Alexander disease, which replicates major features of the human disorder^8^, to perform a forward genetic screen aimed at identification of secreted signalling molecules that promote neuronal death. Our screen recovered Nos, the single fly Nos^16^. We provided strong evidence that NO signalling through cGMP is activated in our fly model, and using both genetics and pharmacology we demonstrated that NO signalling is critical for neuronal death. Importantly, we also showed activation of the same NO signalling pathway in astrocytes of well-characterized mouse models of Alexander disease, as well as upregulation of iNOS in post-mortem brain samples from patients with Alexander disease. Previous experiments have demonstrated that STAT3 can bind to the iNOS promoter and stimulate transcription^44^. Using a well-characterized STAT reporter^20^, we observed upregulation of STAT in GFAP transgenic flies, suggesting that activation of the JAK/STAT pathway could contribute to upregulation of Nos in Alexander disease.

Defining the cell-type-specific effects of soluble signalling molecules *in vivo* has represented a further barrier in understanding the molecular mechanisms of glial-based neurodegeneration. To address cell-type specificity, we have developed a system to independently manipulate gene expression in glia and in neurons in our Alexander disease *Drosophila* model (Fig. 3a). Using the dual-expression system, we provided strong evidence that glial-derived NO activates cGMP signalling in neurons to promote neuronal cell death by reducing the NO receptor, sGC, specifically in neurons. Interestingly, however, we also found that knockdown of sGC in neurons reduces glial cell death as well as neuronal cell death (Fig. 3b). These findings are consistent with signalling, perhaps via NO, from neurons back to glia in our system. A similar amplification process based on release of neuronal NO has been reported in a cell culture model related to experimental autoimmune encephalomyelitis^45^.

Our findings raise the possibility that non-cell autonomous nervous system injury in other ‘gliopathic' disorders might also be driven by glial-derived NO. Indeed, NO has been implicated in a variety of neurological diseases with prominent astrocytic pathology, including Alzheimer's disease and Parkinson's disease^46^. Genetic deletion of iNOS is neuroprotective in experimental models of these disorders^47^,^48^, supporting a functional role for the NO pathway in mediating neuronal pathology. In the current work, we have documented activation of the DNA damage response downstream of NO production in fly and mouse models of Alexander disease. Glia in both *Drosophila* and mouse models showed nuclear accumulation of p53 and evidence of DNA damage, as assayed by development of pH2AX/v-positive foci (Figs 6 and 7). We also found increased levels of p53 in brains from patients with Alexander disease, supporting the relevance of our findings in experimental models to the human disease. Interestingly, astrocytes from patients with Alzheimer's disease also show nuclear accumulation of p53 (ref. 49) and pH2AX^50^, raising the possibility of similar pathways of glial pathogenesis in the most common human neurodegenerative disorder.

In summary, here we have used *Drosophila* genetics, supported by analysis of murine models, to implicate NO as a critical astrocyte-derived neuronal cell death signalling molecule. We have further provided evidence that NO promotes glial dysfunction and death through oxidative stress, DNA damage and p53 activation, well-documented target pathways in astrocytes and other cell types^41^,^51^. Importantly, we used pharmacological inhibition of NO production to support NO signalling as a potential therapeutic target in Alexander disease, and possibly in other gliopathies as well. Indeed, abnormal astrocytes in our Alexander disease models share similarities with astrocytes in Alzheimer's disease and pharmacological inhibition of iNOS with L-NIL provides benefit in a mouse model of Alzheimer's disease^52^,^53^.

## Methods

### *Drosophila* stocks and genetics

All fly crosses were performed at 25 °C; adults were aged at 29 °C to increase transgene expression. The following stocks were obtained from the Bloomington *Drosophila* Stock Center: *repo-GAL4* (glial driver in the GAL4/UAS system), *ET31-QF* (glial driver in the Q system), *elav-GAL4* (neuronal driver in the GAL4/UAS system), *da-GAL4*, *tubP-GAL80*^*ts*^, *UAS-NosRNAi* #1 (TRiP.JF03220), *UAS-lacZ*, *UAS-Gycα99B RNAi* #1 (TRiP.JF03176), *UAS-Gycα99B RNAi* #2 (TRiP.GL01038), *UAS-Gyc*β*100B RNAi* #2 (TRiP.JF03214), *p53*^*11-1B-1*^ *(p53*^*null*^) and *p53 RNAi* (TRiP.JF02153). *UAS-Gyc*β*100B RNAi* #1 (VDRC #100706) was from the Vienna *Drosophila* RNAi Center. Additional stocks used include *Nos*^*Δ15*^ from P. O'Farrell, *hid-lacZ* (*hid*^*20-10*^*-lacZ*) from A. Bergmann, *th-GFP* from J. Jiang, *GstD1-lacZ* from D. Bohmann, *alrm-GAL4* and *mz0709-GAL4* from M. Freeman, *10XStat92E-GFP* from N. Perrimon and *UAS-ATXN3-Q78* from N. Bonini.

*UAS-NosRNAi #2* was generated following the second-generation protocol from the TRiP at the Harvard Medical School by cloning short hairpin targeting *Nos* into the VALIUM20 vector. Genetic Services performed embryo injections. Oligo sequences were as follows: 5′- TCGCAGCATTTCACATCGATA -3′ (sense sequence), 5′- TATCGATGTGAAATGCTGCGA -3′ (antisense sequence). QUAS-GFAP^R79H^ was constructed by digesting both UAS-GFAP^R79H^ construct^8^ and the pQUAST vector^34^ with EcoRI and NotI, followed by subcloning of the GFAP^R79H^ fragment into the pQUAST vector. BestGene performed embryo injections.

### Transgenic mice

Five-month-old male GFAP^Tg^ (Tg73.7) mice^6^ were used for western blot and 3-month-old GFAP^+/R236H^ mice^7^ and GFAP^Tg^ (Tg73.7)^6^ mice, both male and female mice in the FVB/N background, were used for immunofluorescence staining. Same-sex age-matched wild-type littermates were used as controls. All procedures were approved by the Institutional Animal Care and Use Committee of the Graduate School of the University of Wisconsin-Madison. Tauopathy transgenic mice rTg(tau_P301L_)4510 (ref. 39) were 7 months old; controls were age-matched littermates not carrying the human tau transgene.

### Drug feedings

L-NAME (Cayman chemical) and D-NAME (Sigma) were dissolved in water at 5 and 10 mM (Fig. 2c,d) and then mixed with instant *Drosophila* medium (Carolina Biological). Newly eclosed flies were fed on drug-embedded food for a total of 15 days for TUNEL analysis and 3 days for seizure quantification. Flies were transferred to new drug-embedded food every 3 days.

### Behavioural analysis

Flies were collected under CO_2_ anaesthesia at 1 day after eclosion and kept at five animals per vial for 1 day, without further anaesthesia, before analysis. Drug-fed flies were kept on drug-embedded food for 3 days before analysis. For testing, vials were mechanically stimulated on a VWR mini vortexer for 10 s at maximum speed. Seizures were defined as repetitive contractions of legs or wings, or episodes of paralysis lasting more than 1 s (refs 8, 54). Seizure frequency was calculated by dividing the number of flies with seizures by the total number of flies tested. Statistical significance was evaluated using the *χ*^2^-test. Each data point represents seizure frequency (% of flies with seizures).

### Immunostaining and TUNEL analysis

For tissue sections, adult flies were fixed in formalin and embedded in paraffin. Four-micrometre serial frontal sections were prepared through the entire fly brain and placed on a single glass slide. In some studies, whole-mount *Drosophila* brain preparations were alternatively used. Mouse brains were fixed in 4% paraformaldehyde, embedded in paraffin and sectioned at a thickness of 6 μm.

For immunostaining, paraffin slides were processed through xylene, ethanol and into water. Antigen retrieval by boiling in sodium citrate, pH 6.0, was performed before blocking. Slides were blocked in PBS containing 0.3% Triton X-100 and 2% milk for 1 h and then incubated with appropriate primary antibodies overnight. Antibodies are summarized in Supplementary Table 1. For immunohistochemistry, biotin-conjugated secondary antibodies (1:200, SouthernBiotech) and the avidin-biotin-peroxidase complex (Vectastain Elite, Vector Laboratories) staining was performed using DAB (Vector Laboratories) as a chromagen. For double-labelling studies, secondary antibodies coupled to Alexa 488 or Alexa 555 (1:200, Invitrogen) were used. All immunostaining data were replicated in at least three animals, and representative images are shown.

As a control, diluted polyclonal nitrotyrosine antibody^22^,^23^,^24^ was incubated with nitrated BSA (Upstate; final concentration 1 mg ml^−1^) for 2 h at room temperature before being applied to tissue; the control was incubated with non-nitrated BSA (final concentration 1 mg ml^−1^). To reduce nitrotyrosine to aminotyrosine, tissue was incubated with 10 mM or 50 mM dithiothreitol diluted in PBS for 10 min at 100 °C; the control was incubated with PBS^36^. A dose-dependent reduction in immunoreactivity was observed (Supplementary Fig. 4b).

Apoptotic cell death was visualized using TUNEL labelling according to the manufacturer's instructions (TdT FragEL DNA fragmentation kit, Calbiochem), with an additional avidin-biotin-peroxidase amplification step. The number of TUNEL-positive cells was counted by examining serial frontal sections (4 μm) of the entire brains from at least six animals per genotype or condition. Fluorescent TUNEL labelling was performed with Alexa 488-conjugated streptavidin (Invitrogen). Data are presented as both cell counts (Figs 2a,c and 3b) and fold changes (Supplementary Figs 2a,i and 3g) to facilitate assessment of the magnitude of the changes observed. Statistical analysis was performed using one-way analysis of variance (ANOVA) with Tukey's multiple comparison test. Each data point represents the mean±s.e.m. The nuclear morphology of TUNEL-positive cells (Supplementary Fig. 2d) was evaluated with 4,6-diamidino-2-phenylindole (DAPI) staining in six GFAP transgenic flies.

Quantification of the number of β-gal-positive cells for *hid-lacZ* activation, the number of GFP-positive cells for *th-GFP* activation and the number of cleaved Dcp-1-positive cells (Supplementary Fig. 2e–g) was performed by examining serial frontal sections (4 μm) of the entire brains from at least six animals per genotype.

Quantification of the number of β-gal-positive cells for *GstD1-lacZ* activation (Fig. 5b) and the number of p53-positive cells (Fig. 6h) was performed by counting the positive cells in one complete optic lobe from at least six animals per genotype. Statistical analysis was performed using one-way ANOVA with Tukey's multiple comparison test. Each data point represents the mean±s.e.m.

### NADPH-diaphorase staining

The NADPH-diaphorase staining protocol was adapted from Ott and Elphick^55^. Briefly, brains were dissected in PBS and fixed in freshly mixed methanol and formalin (1:1) for 2 h on ice, followed by rinsing in PBS. Brains were then incubated in a PBS solution containing 0.3% Triton X-100, 0.5 mM NADPH (Sigma) and 0.2 mM NBT (Fisher). Incubations were carried out first in a cold room and then at room temperature until a proper blue developed. Brains were rinsed with PBS and images were acquired using a SPOT camera. Six brains for each genotype were analysed. Each data point represents the mean±s.e.m.

### NO measurement

The NO chemical sensor FL2E (Strem Chemicals) was dissolved in dimethylsulphoxide (DMSO) and mixed with CuCl_2_ immediately before use to form Cu_2_(FL2E)^19^. Brains were dissected in PBS and added to 10 μM Cu_2_(FL2E). Time-lapse imaging was performed for each brain on a SPOT camera with an interval of 2 min for a total of 10 min. Seven brains for each genotype were dissected and imaged. ImageJ was used to quantify fluorescence intensity, and two-way ANOVA with the Bonferroni post test was used for statistical analysis. Each data point represents the mean±s.e.m.

### cGMP measurement

A single fly head was homogenized in sample buffer and then centrifuged at 600*g* for 15 min at 4 °C. The supernatant was used for determination of cGMP concentration following the manufacturer's instructions (aCella-cGMP immunoassay, Cell Technology). Each data point represents the mean±s.e.m.

### Western blots

For standard western blot analysis, adult *Drosophila* heads were homogenized in 1 × Laemmli buffer (Sigma). For murine samples, cortex was homogenized in homogenization buffer (50 mM Tris, 150 mM NaCl, pH 7.4, with protease inhibitor cocktail, Thermo Scientific) and centrifuged at 100,000*g* for 1 h at 4 °C. The supernatant was used for western blot. For human brains, grey matter from the frontal cortex was homogenized in homogenization buffer and centrifuged at 100,000*g* for 1 h at 4 °C. The supernatant was used for western blots. For nitrated BSA (Upstate), serial dilutions were made in PBS and then added to Laemmli buffer (Sigma) at the indicated concentrations (Supplementary Fig. 4b). Samples were boiled for 10 min at 100 °C, briefly centrifuged and subjected to SDS–polyacrylamide gel electrophoresis using 10% or 4–12% gels (Lonza). Proteins were transferred to nitrocellulose membranes (Bio-Rad), blocked in 2% milk in PBS with 0.05% Tween-20 and immunoblotted with primary antibodies (Supplementary Table 1). The appropriate anti-mouse or anti-rabbit horseradish peroxidase-conjugated secondary antibody (1:50,000, SouthernBiotech) was applied and the signal was detected with West Femto chemiluminescent substrate (Thermo Scientific). All blots were repeated at least three times, and representative blots are presented in the figures. Full-length images of cropped blots are shown in Supplementary Figs 6 and 7. Western blots were quantified with NIH Image J software, and statistical analysis was performed using the unpaired *t*-test (two samples) or one-way ANOVA with Tukey's multiple comparison test (three or more samples). Each data point represents the mean±s.e.m.

### Human samples

Frozen frontal cortex from five controls (mean age 10 years, range 1–28 years; 2 females and 3 males), five Alexander disease patients (mean age 13 years, range 1–27 years; 2 females and 3 males) and four metachromatic leukodystrophy patients (mean age 10 years, range 3–21 years; 2 females and 2 males) were obtained from the NICHD Brain and Tissue Bank for Developmental Disorders at the University of Maryland, Baltimore, MD. Informed consent for tissue distribution was obtained by the NICHD Brain and Tissue Bank. The studies were performed under IRB-approved protocol 2003P000813 from Partners Healthcare to M.B. Feany. GFAP mutations in the Alexander disease patients included R79C, R239H (2 cases), K63E and L359V. All cases had typical neuropathology of Alexander disease, including multiple Rosenthal fibres. Post-mortem intervals were comparable between cases and controls and were less than 24 h in all cases. For immunostaining analysis, tissue was thawed and fixed in 4% paraformaldehyde overnight before paraffin embedding.

### Confocal microscopy

All the fluorescent images were taken as Z-stacks on a confocal microscope (a Zeiss LSM 510 or a Leica SP8 X confocal microscope at Harvard NeuroDiscovery Center Enhanced Neuroimaging Core facility or an Olympus Fluoview 1000 confocal microscope at Harvard Medical School Neurobiology Imaging facility, NINDS P30 Core Center grant #NS072030) and visualized in two-dimensional projections of Z-stacks. Control and experimental samples were imaged with the same laser setting and the same Z-stack thickness.

### Real-time PCR

RNA was isolated from *Drosophila* head homogenates using QIAzol (Qiagen) and reverse-transcribed using the High-Capacity cDNA Reverse Transcription kit (Applied Biosystems) according to the manufacturer's instruction. Real-time PCR was performed and monitored using SYBR Green PCR Master Mix (Applied Biosystems) in a StepOnePlus Real-Time PCR system (Applied Biosystems) according to the manufacturer's instructions. *Drosophila* ribosomal protein *RpL32* was used as a control. Each data point represents the mean±s.e.m.

Primers:

*Gycα99B*: 5′- GTGGCCAAGTACCGCCAGA -3′; 5′- CGCTGGCCTCGTCCATCT -3′

*Gycβ100B*: 5′- AATCGTTCAGGCCGGCA -3′; 5′- CGAAGTTGAGCTGCAGGTGC -3′

*Rpl32*: 5′- GACCATCCGCCCAGCATAC -3′; 5′- CGGCGACGCACTCTGTT -3′-

### Statistical analysis

GraphPad Prism (v5) was used to perform two-tailed unpaired Student's *t*-tests (for two groups), one-way ANOVA with Tukey's *post hoc* tests (for three groups or more) and two-way ANOVA with Bonferroni's post test (for two variables). GraphPad QuickCalcs (a web tool) was used to perform a *χ*^2^-test (for comparison between observed frequency and expected frequency). *P*<0.05 was considered statistically significant. No statistical methods were used to predetermine sample sizes; sample sizes were similar to those reported in prior publications. Variance within each group was not calculated, but assumed to be similar. All photomicrographs shown (immunolabelling, light microscopy and blots) are representative of at least three biological replicates. All quantitative data are presented as mean±s.e.m., except for seizure frequency (percentage of flies with seizures). Data for all experiments were collected and processed randomly, but no formal randomization was carried out. Data collection and analysis were not performed blind given the nature of the experiments performed.

## Additional information

**How to cite this article:** Wang, L. *et al*. Nitric oxide mediates glial-induced neurodegeneration in Alexander disease. *Nat. Commun.* 6:8966 doi: 10.1038/ncomms9966 (2015).

## Supplementary Material

Supplementary Information Supplementary Figures 1-9 and Supplementary Table 1
