# Supplementary material for: Nitric oxide mediates glial-induced neurodegeneration in Alexander disease
Source: Nat Commun. 2015 Nov 26;6:8966. doi: 10.1038/ncomms9966 (PMC4674772; doi:10.1038/ncomms9966)
Supplement: Supplementary Information — Supplementary Figures 1-9 and Supplementary Table 1 [file ncomms9966-s1.pdf]

Supplementary figure-1 Feany

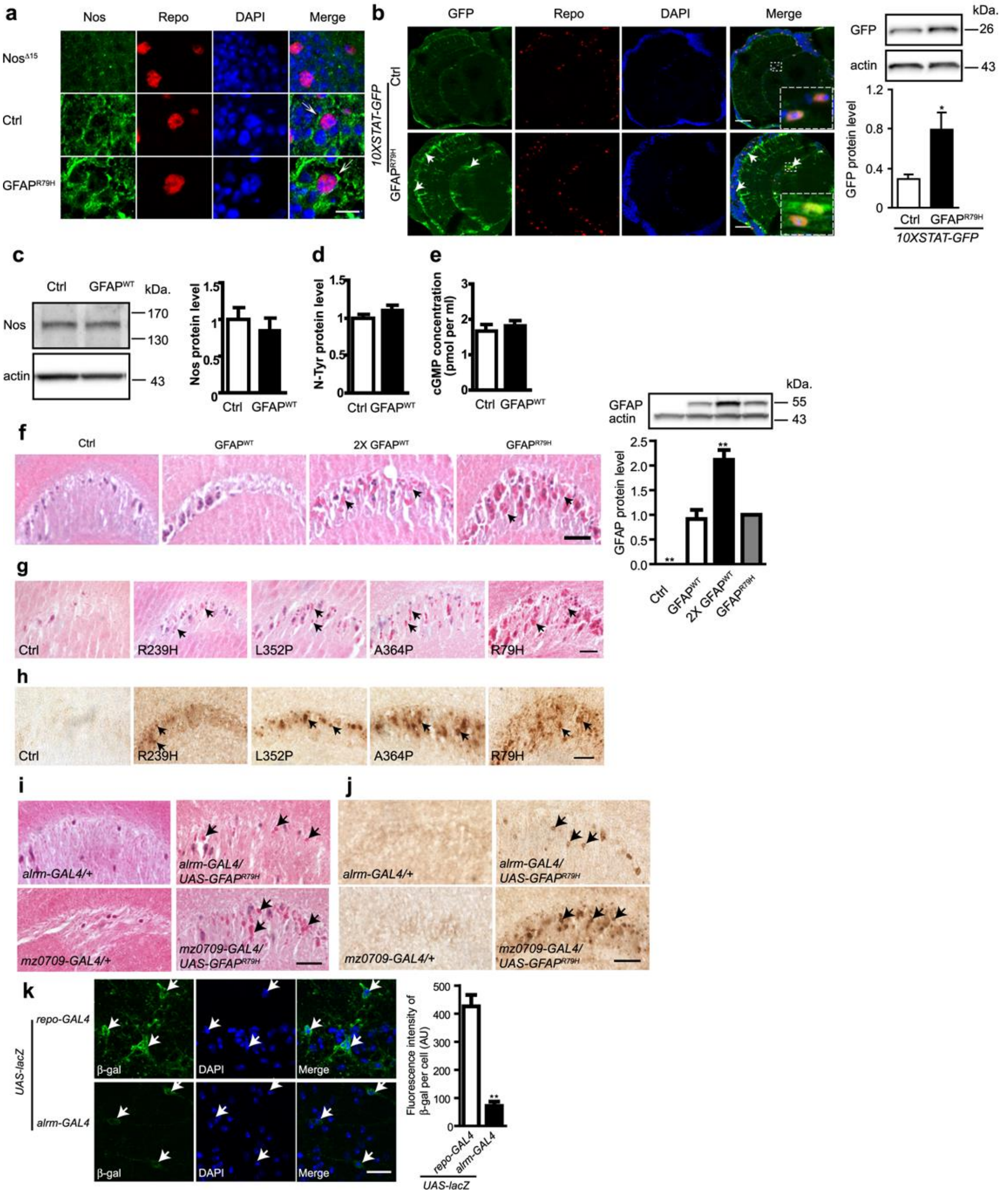

Supplementary Figure 1. (**a**) Nos is detected in glial cells in both control and GFAP^{R79H} transgenic flies (arrows), but not in deletion mutant *Nos*$^{\Delta 15}$ animals. Repo is a glial cell marker. DAPI labels nuclei. Flies were 20 days old. Scale bar is 5 µm. Ctrl is

repo-GAL4/+. GFAP^{R79H} is *repo-GAL4, UAS-GFAP^{R79H}/+*. (**b**) Immunofluorescence (left) and western blotting (right) demonstrate increased STAT expression in GFAP transgenic flies compared to age-matched controls. Ctrl is *repo-GAL4/+*. GFAP^{R79H} is *repo-GAL4, UAS-GFAP^{R79H}/+*. Flies were 20 days old. Scale bar: 25 μm. *p=0.0267, t=2.917 df=6; unpaired t-test, n=4. (**c**) Western blot shows similar levels of Nos in control (Ctrl) and GFAP^{WT} transgenic flies. The blot was reprobed for actin to illustrate equivalent loading. p=0.5673, t=0.6050 df=6; unpaired t-test, n=4 brains per genotype. (**d**) Nitrotyrosine (N-tyr) modified protein levels were equal in control (Ctrl) and GFAP^{WT} transgenic flies. p=0.2274, t=1.286 df=10; unpaired t-test, n=6 brains per genotype. (**e**) The level of cGMP was not increased in GFAP^{WT} transgenic flies as assayed by chemiluminescent immunoassay. p=0.5022, t=0.6961 df=10; unpaired t-test, n=6 brains per genotype. In figures **c**-**e**, flies were 20 days old. Control (Ctrl) is *repo-GAL4/+*. GFAP^{WT} is *repo-GAL4, UAS-GFAP^{WT}/+*. (**f**) H&E staining reveals robust Rosenthal fiber-like inclusion formation only when wild type human GFAP is present at increased levels (2X GFAP^{WT}), but not when GFAP^{WT} is expressed at equivalent levels to GFAP^{R79H} (arrows). Scale bar: 10 microns. n=3 for western blot quantification, **p<0.0001, F=40.31 df=11, all compared to mutant GFAP, one-way ANOVA. Flies were 20 days old. Ctrl: *repo-GAL4/+*; GFAP^{WT}: *repo-GAL4, UAS-GFAP^{WT}/+*; 2x GFAP^{WT}: *repo-GAL4, UAS-GFAP^{WT}/UAS-GFAP^{WT}*; GFAP^{R79H} is *repo-GAL4, UAS-GFAP^{R79H}/+*. (**g**) H&E staining reveals Rosenthal fiber-like inclusions (arrows) in flies expressing Alexander disease-associated mutant forms of GFAP. Flies were 20 days old. Scale bar is 10 μm. Ctrl: *repo-GAL4/+*. Driver:

repo-GAL4/+. (**h**) GFAP immunohistochemistry highlights aggregates (arrows) in GFAP transgenic flies. Flies were 1 day old. Scale bar is 10 µm. Ctrl: *repo-GAL4/+*. Driver: *repo-GAL4/+.* (**i**) H&E staining reveals Rosenthal fiber-like inclusions in $GFAP^{R79H}$ transgenic flies with expression driven by the glial subtype drivers *alrm-GAL4* (astrocyte-like glia, top panel, arrows) and *mz0709-GAL4* (ensheathing glia, bottom panel, arrows). Flies were 20 days old. Scale bar is 10 µm. (**j**) GFAP immunohistochemistry highlights aggregates in transgenic flies expressing $GFAP^{R79H}$ in glial subtypes using *alrm-GAL4* (top panel, arrows) and *mz0709-GAL4* (bottom panel, arrows). Flies were 1 day old. Scale bar is 10 µm. (**k**) Immunofluorescence quantification demonstrated less β-galactosidase expression per astrocyte-like glial cell driven by *alrm-GAL4* driver than *repo-GAL4* driver. Quantification was performed on 6 brains from each genotype, with 12 cells from a similar region in each brain. Each data point represents the mean ± SEM. **$p<0.0001$, $t=8.169$ $df=10$, unpaired t-test. AU: artificial unit.

Supplementary figure-2 Feany

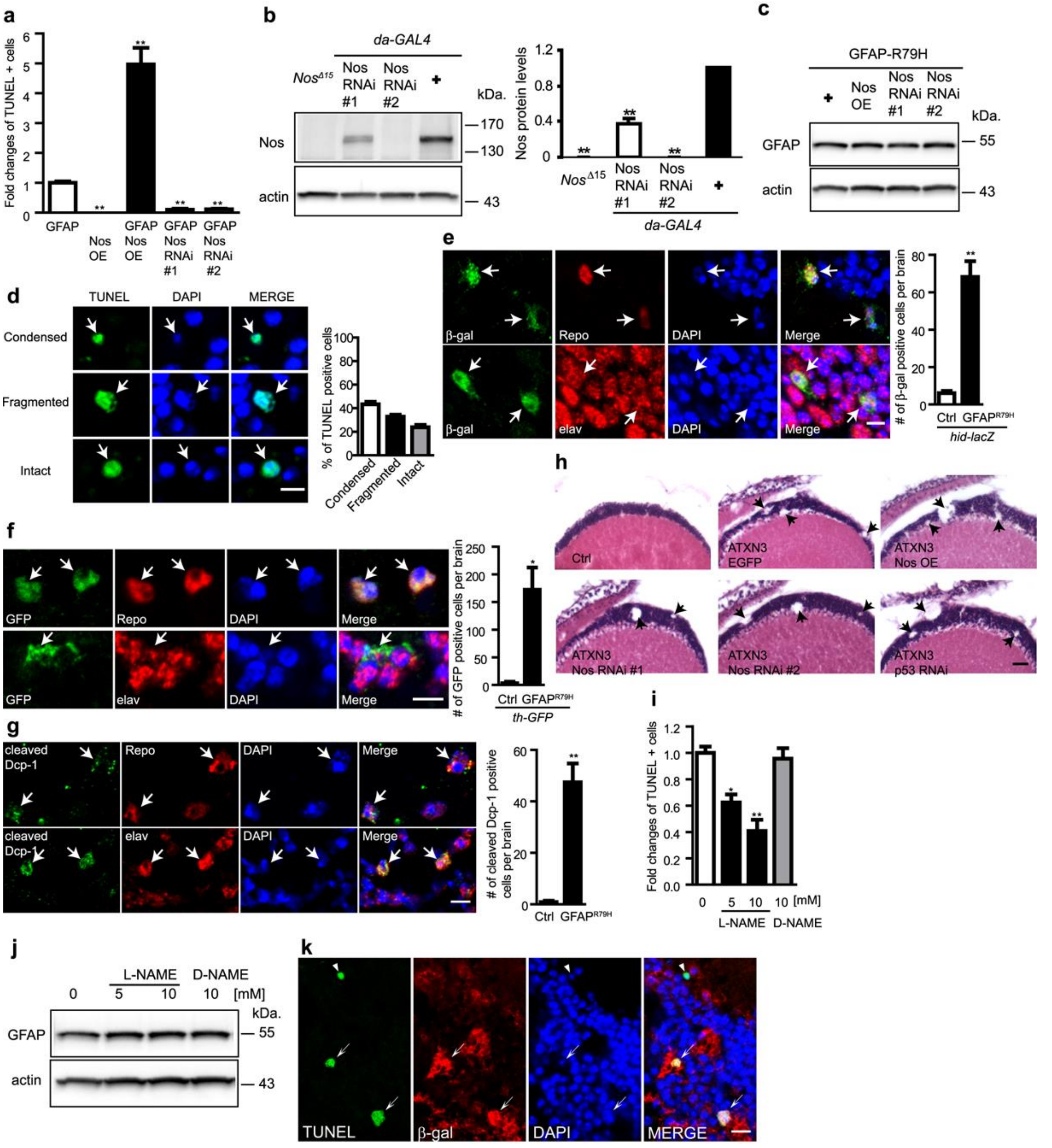

Supplementary Figure 2. (**a**) Analysis of fold changes showed a 4-fold increase of TUNEL-positive cells in GFAP transgenic flies when Nos is overexpressed (GFAP/Nos OE) and a 90% decrease when Nos expression is reduced (GFAP/Nos RNAi). **p<0.0001, F=72.53 df=29, one-way ANOVA with Tukey's multiple comparison test; n=6

per genotype. Flies were 20 days old. Genotypes are indicated in the figure label. Driver: *repo-GAL4*. (**b**) Nos protein levels are significantly reduced in 2 independent transgenic *Nos* RNAi lines using the ubiquitous *da-GAL4* driver. The blot was reprobed for actin to illustrate equivalent loading. ** $p<0.0001$, compared to *da-GAL4/+*, $F=247.6$ $df=19$; one-way ANOVA with Tukey's multiple comparison test, n=5 brains per genotype. Flies were 1 day old. The deletion mutant $Nos^{\Delta 15}$ was used as negative control. Genotypes are indicated in the figure label. +: *da-GAL4/+*. (**c**) Western blot demonstrates equivalent GFAP levels in $GFAP^{R79H}$ transgenic flies alone and in combination with *Nos* modifiers. The blot was reprobed for actin to illustrate equivalent loading. Flies were 1 day old. Genotypes are indicated in the figure label. Driver: *repo-GAL4*. +: *repo-GAL4, UAS-*$GFAP^{R79H}$*/+*. (**d**) Immunofluorescence revealed condensed (43.36% ± 2.06%, middle panel) and fragmented (32.82% ± 1.64%, bottom panel) nuclei morphologies in TUNEL-positive cells. Flies were 20 days old. Scale bar is 3 µm. Genotype: *repo-GAL4, UAS-*$GFAP^{R79H}$*/+*. n=6 brains were used for quantification. (**e**) Double label immunofluorescence of β-galactosidase and cell type specific markers demonstrated activation of the *hid-lacZ* in both glial (Repo, top panel) and neuronal cells (elav, bottom panel) in GFAP transgenic flies. Quantification is shown in the right panel. Ctrl: *repo-GAL4/hid-lacZ*. Flies were 20 days old. ** $p<0.0001$, n=6 per genotype, $t=7.369$ $df=10$, unpaired t-test. Scale bar is 3 µm. (**f**) Double label immunofluorescence of GFP and cell type specific markers demonstrated activation of *th-GFP* in both glial (Repo, top panel) and neuronal cells (elav, bottom panel) in GFAP transgenic flies. Quantification

was shown in the right panel. Ctrl: *repo-GAL4/th-GFP*. Flies were 20 days old. * p=0.0026, n≥6 per genotype, t= 3.865 df=11, unpaired t-test. Scale bar is 3 μm. (**g**) Double label immunofluorescence demonstrated activation of cleaved Dcp-1 in both glial (Repo, top panel) and neuronal cells (elav, bottom panel) in GFAP transgenic flies. Quantification is shown in the right panel. Ctrl: *repo-GAL4/+*. Flies were 20 days old. ** p<0.0001, n≥6 per genotype, t=5.822 df=11, unpaired t-test. Scale bar is 3 μm. (**h**) H&E staining shows that Nos and p53 modifiers do not markedly alter neurodegeneration (arrows, vacuolation) in mutant human ataxin 3 transgenic flies. ATXN3 represents *repo-Gal4, tub-Gal80^{ts}/UAS-ATXN3-Q78.* Ctrl: *repo-GAL4, tub-Gal80^{ts}/+*. Flies were 7 days old. Scale bar is 10 μm. (**i**) Analysis of fold changes revealed a 50% reduction of TUNEL-positive cells in GFAP transgenic flies with administration of Nos inhibitor L-NAME. *p<0.01, **p<0.001, F=18.85 df=26, n≥6 per condition, one-way ANOVA with Tukey's multiple comparison test. Flies were 15 days old. Genotype: *repo-GAL4, UAS-GFAP^{R79H}/+*. (**j**) Western blot shows equivalent GFAP levels in control and drug-fed flies (**Fig. 2c,d**). The blot was reprobed for actin to illustrate equivalent loading. Genotype: *repo-GAL4, UAS-GFAP^{R79H}/+*. Flies were 3 days old. (**k**) Double label immunofluorescence for TUNEL and β-galactosidase marks glial apoptotic cells (arrows) in GFAP^{R79H} transgenic flies. The arrowhead indicates a TUNEL-positive, β-gal negative neuron. DAPI labels nuclei. Flies were 20 days old. Scale bar is 5 μm. Genotype: *UAS-lacZ/+; repo-GAL4, UAS-GFAP^{R79H}/+*.

Supplementary figure-3 Feany

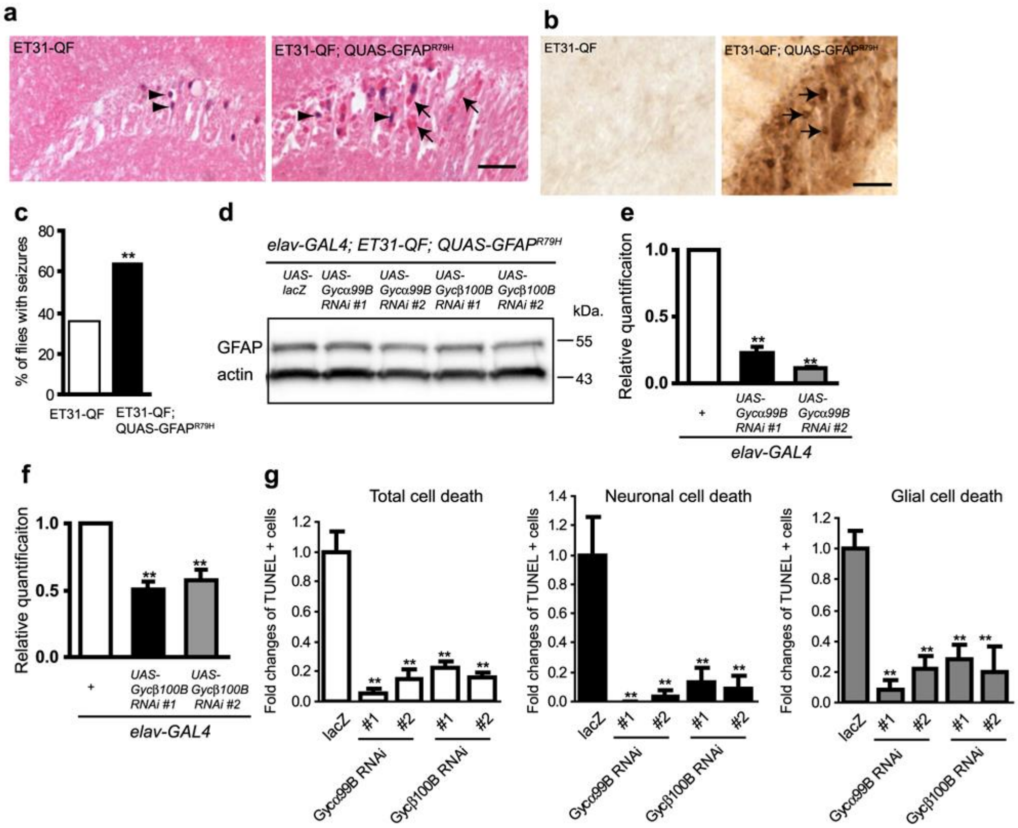

Supplementary Figure 3. (**a**) H&E staining reveals the formation of Rosenthal fiber-like inclusions (arrows) when GFAP^{R79H} (*QUAS-GFAP*^{R79H}) was expressed in glia with the Q system glial-specific driver *ET31-QF*. Arrowheads indicate nuclei marked by blue hematoxylin staining. Flies were 20 days old. Scale bar is 10 µm. (**b**) GFAP immunohistochemistry shows aggregates (arrows) when GFAP^{R79H} (*QUAS-GFAP*^{R79H}) was expressed in glia with the Q system glial-specific driver *ET31-QF*. Flies were 1 day old. Scale bar is 5 µm. (**c**) Seizure frequency is increased in GFAP^{R79H} (*QUAS-GFAP*^{R79H}) transgenic flies with *ET31-QF* as driver. **$p<0.0001$, $\chi^2=35.472$ df=1; chi-square test, n>100 per genotype. Flies were 1 day old. (**d**) Western blot demonstrates equivalent GFAP levels in GFAP^{R79H} transgenic flies with RNAi modifiers that reduce sGC levels in

the dual transcriptional expression system (**Fig. 3b**). The blot was reprobed for actin to illustrate equivalent loading. Flies were 1 day old. Genotypes are indicated in the figure label. (**e**) RT-PCR shows reduced mRNA levels for the alpha subunit of sGC in two transgenic RNAi lines, *UAS-Gyc*α*99B RNAi #1* and *UAS-Gyc*α*99B RNAi #2*, using the *elav-GAL4* driver. *Rpl32* was used as a control. ** p<0.0001, F=328.1 df=8; one-way ANOVA with Tukey's multiple comparison test, n=3. Flies were 1 day old. Genotypes are indicated in the figure label. +: *elav-GAL4/+*. (**f**) RT-PCR shows reduced mRNA levels for the beta subunit of sGC in two transgenic RNAi lines, *UAS-Gyc*β*100B RNAi #1* and *UAS-Gyc*β*100B RNAi #2*, using the *elav-GAL4* driver. *Rpl32* was used as a control. ** p=0.0018, F=21.87 df=8; one-way ANOVA with Tukey's multiple comparison test, n=3. Flies were 1 day old. Genotypes are indicated in the figure label. +: *elav-GAL4/+*. (**g**) Analysis of fold changes of TUNEL-positive cells in the dual system showed that reducing expression of sGC receptors in neurons with RNAi lines rescued 70-80% glial and 80-90% neuronal cell death in GFAP transgenic flies. **p<0.0001, compared to *UAS-lacZ* control. one-way ANOVA with Tukey's multiple comparison test, n=6 per genotype. Flies were 20 days old. Genotype: *elav-GAL4; ET31-QF; QUAS-GFAP*^{R79H}*/UAS-RNAi*. RNAi lines are indicated in the figure label.

Supplementary figure-4 Feany

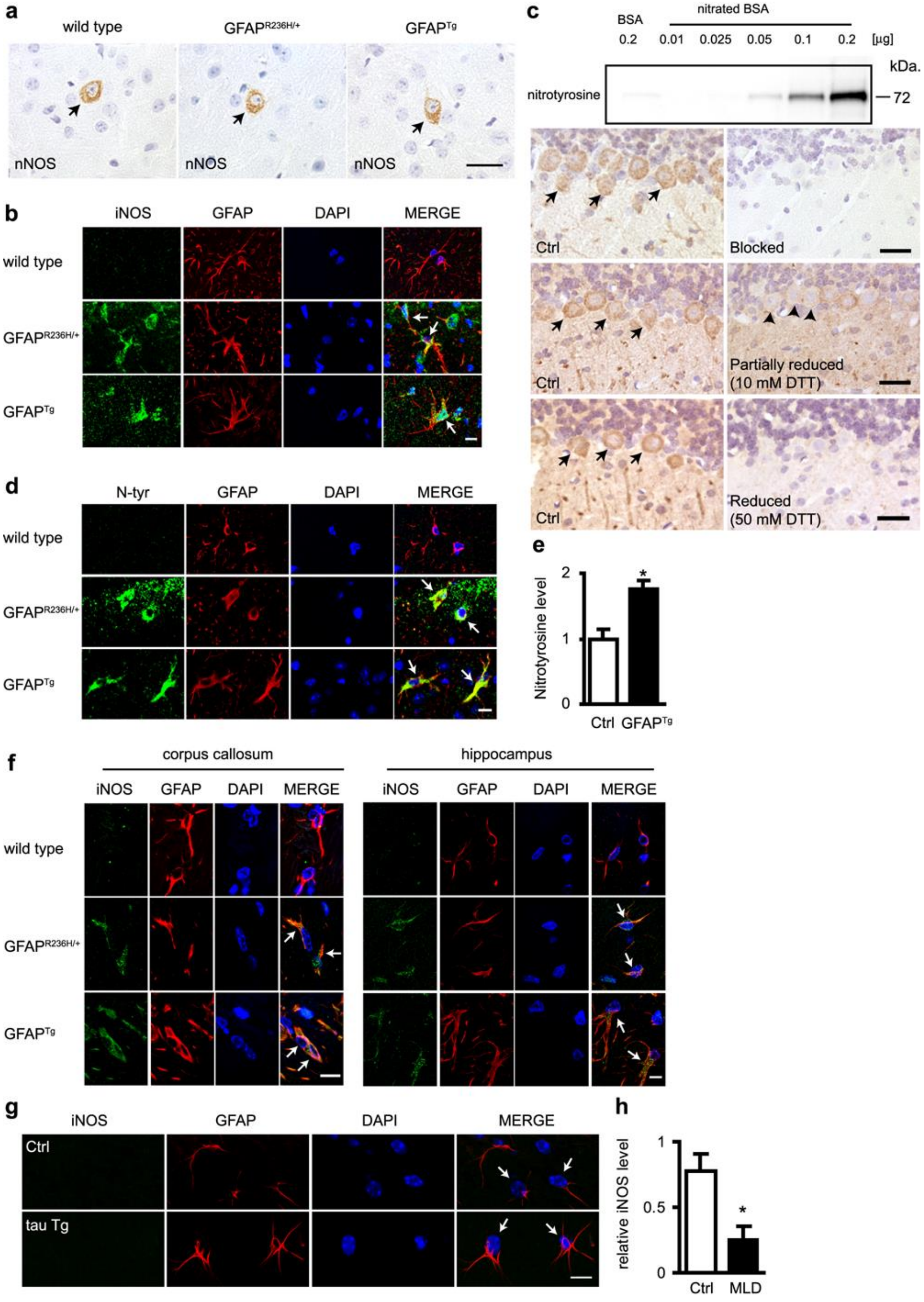

Supplementary Figure 4. (a) Immunohistochemical staining detects no difference of nNOS expression between wild type and 3-month-old GFAP$^{R236H/+}$ and GFAP^{Tg} mice

(arrows). Scale bar is 50 µm. **(b)** Double label immunofluorescence with a monoclonal antibody to iNOS demonstrates increased expression of iNOS in astrocytes of 3-month-old $GFAP^{R236H/+}$ and $GFAP^{Tg}$ mice (arrows), but not in age-matched wild type littermate controls. GFAP was used to identify astrocytes. DAPI labels nuclei. Scale bar is 10 µm. (**c**) The specificity of the polyclonal anti-nitrotyrosine antibody was validated in immunoblot by detection of nitrated BSA but not non-nitrated BSA (top panel); additionally, blocking nitrotyrosine antibody with nitrated BSA (1 mg/ml) for 2 hours at room temperature (top panel) or reducing nitrotyrosine to aminotyrosine with 50 mM DTT on tissue sections (bottom panel). 10 mM DTT partially converted nitrotyrosine (middle panel, arrowheads). Scale bar is 20 µm. (**d**) Double label immunofluorescence with a monoclonal antibody to nitrotyrosine proteins (N-tyr) demonstrates increased expression of nitrotyrosine proteins in astrocytes of 3-month-old $GFAP^{R236H/+}$ and $GFAP^{Tg}$ mice (arrows), but not in age-matched wild type littermate controls. GFAP was used to identify astrocytes. DAPI labels nuclei. Scale bar is 10 µm. (**e**) Quantification of nitrotyrosine proteins with a monoclonal antibody reveals a significant increase in $GFAP^{Tg}$ mice (5-month-old) compared to wild type littermate controls. *p=0.0161, t=4.003 df=4; unpaired t-test, n=3 per genotype. Ctrl is age-matched wild type littermates. (**f**) Double label immunofluorescence detects increased iNOS expression in astrocytes at corpus callosum and hippocampus (arrows), but not in age-matched wild type littermate controls. GFAP was used to identify astrocytes. DAPI labels nuclei. Scale bar is 10 µm. (**g**) iNOS expression was not detected in astrocytes of 7-month-old human tau transgenic mice

(bottom panel, arrows), or in controls (top panel, arrows). Control (Ctrl) is age-matched littermate not carrying the human tau transgene mice. Scale bar is 10 µm. (**h**) Quantification of iNOS protein levels reveals a decrease in metachromatic leukodystrophy (MLD) patients compared to control. *p=0.0184, t=3.057 df=7; unpaired t-test, n=5 for ctrl and 4 for MLD.

Supplementary figure-5 Feany

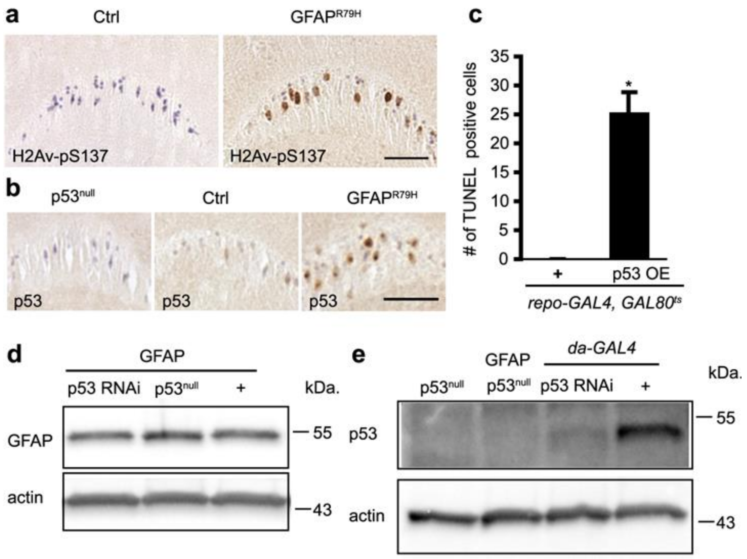

Supplementary Figure 5. (**a**-**b**) Immunohistochemical staining reveals increased H2Av-pS137 (**a**) and p53 (**b**) in GFAP^{R79H} transgenic flies, but not in controls. Flies were 20 days old. Scale bar is 20 µm. Control (Ctrl) is *repo-GAL4/+*. GFAP^{R79H} is *repo-GAL4, UAS-GFAP*^{R79H}/+. (**c**) Overexpression of p53 (p53 OE) in *Drosophila* glia induces cell death. Flies were 20 days old. * p=0.0001, t=6.5661 df=9; unpaired t-test, n=6 per genotype. +: *repo-GAL4, tubP-GAL80*^{ts}/+. p53 OE: *UAS-p53/+; repo-GAL4, tubP-GAL80*^{ts}*/+.* (**d**) Western blot demonstrates equivalent GFAP levels in GFAP^{R79H} transgenic flies alone and in combination with p53 modifiers. The blot was reprobed for actin to illustrate equivalent loading. Flies were 1 day old. Genotypes are indicated in the figure label. Driver: *repo-GAL4*. +: *repo-GAL4, UAS-GFAP*^{R79H}/+. (**e**) No p53 is detected in *p53*^{null} animals; p53 levels are markedly reduced by expression of transgenic RNAi using the ubiquitous *da-GAL4* driver. The blot was reprobed for actin to illustrate equivalent loading. Flies were 1 day old. Genotypes are indicated in the figure label.

Driver is *repo-GAL4* in the second lane. +: *da-GAL4/+*.

Supplementary figure-6 Feany

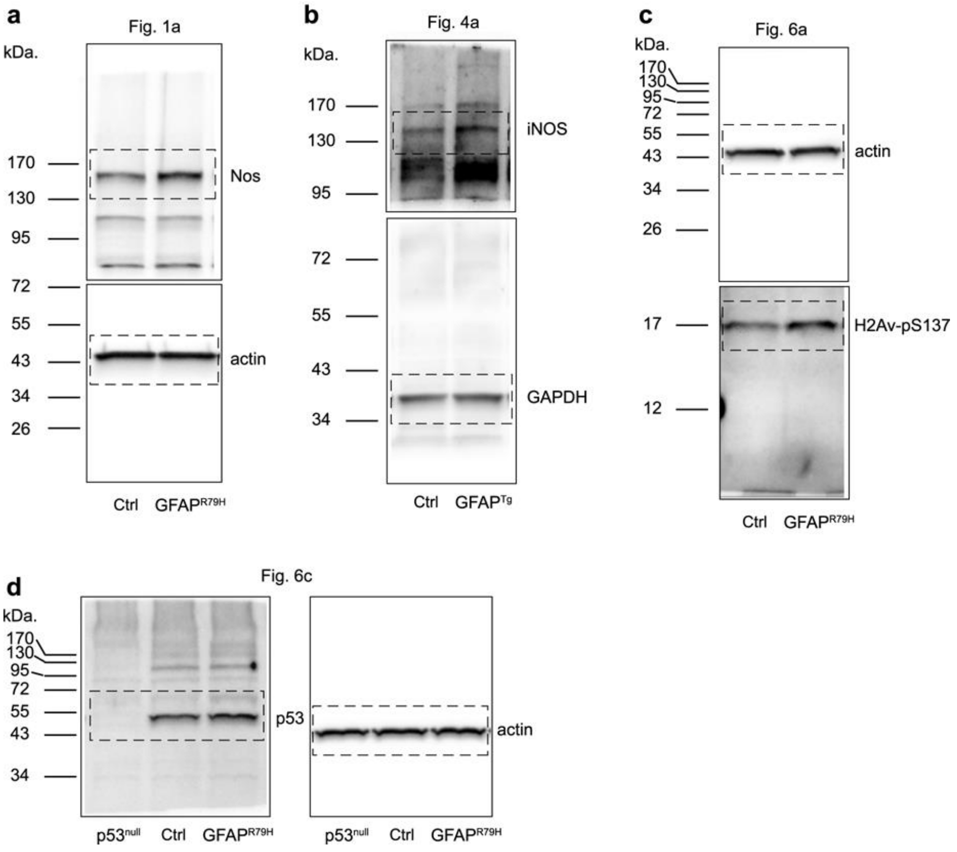

Supplementary Figure 6. Full length images of blots from the main text.

Supplementary figure-7 Feany

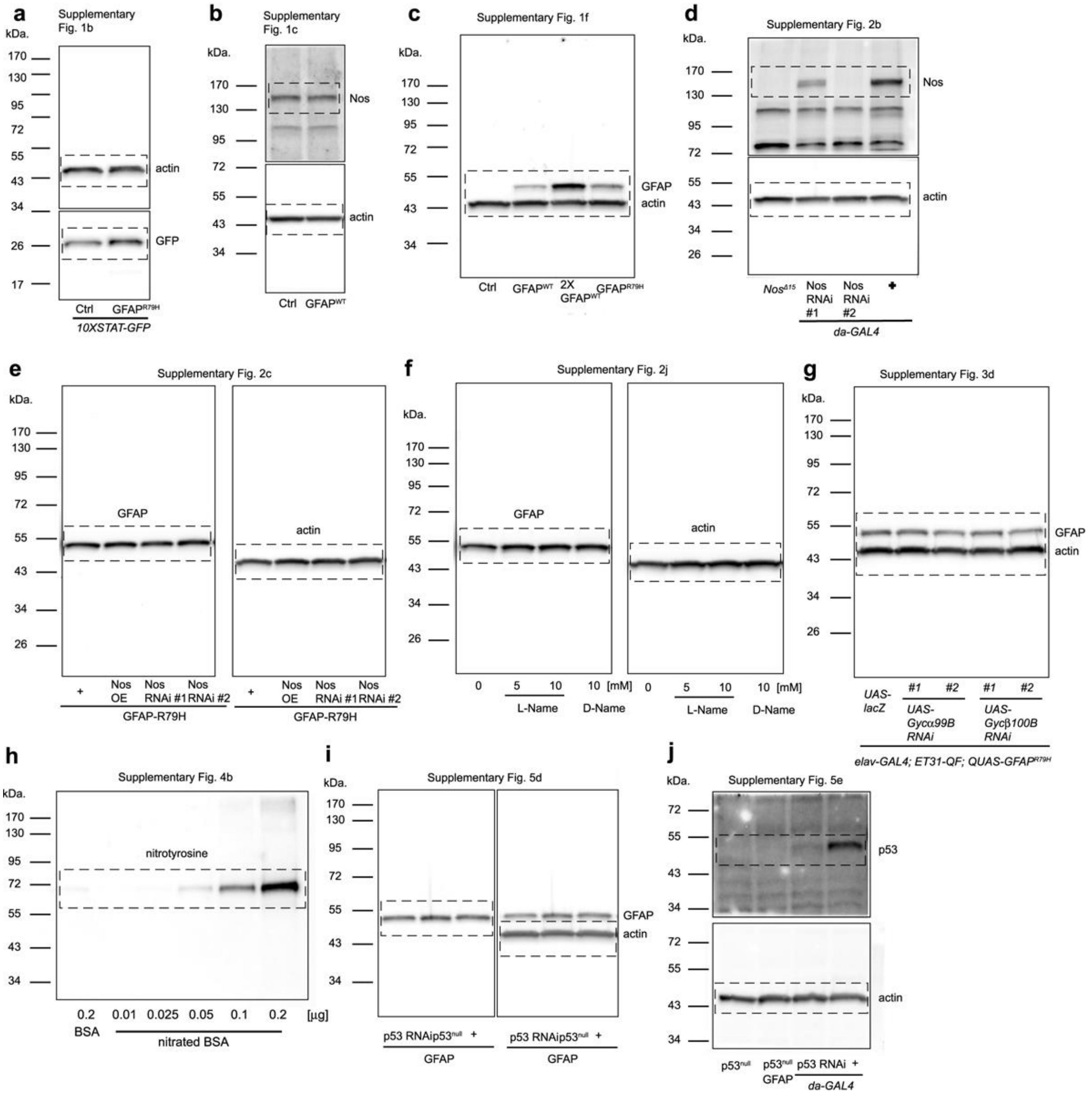

Supplementary Figure 7. Full length images of blots from supplementary figures.

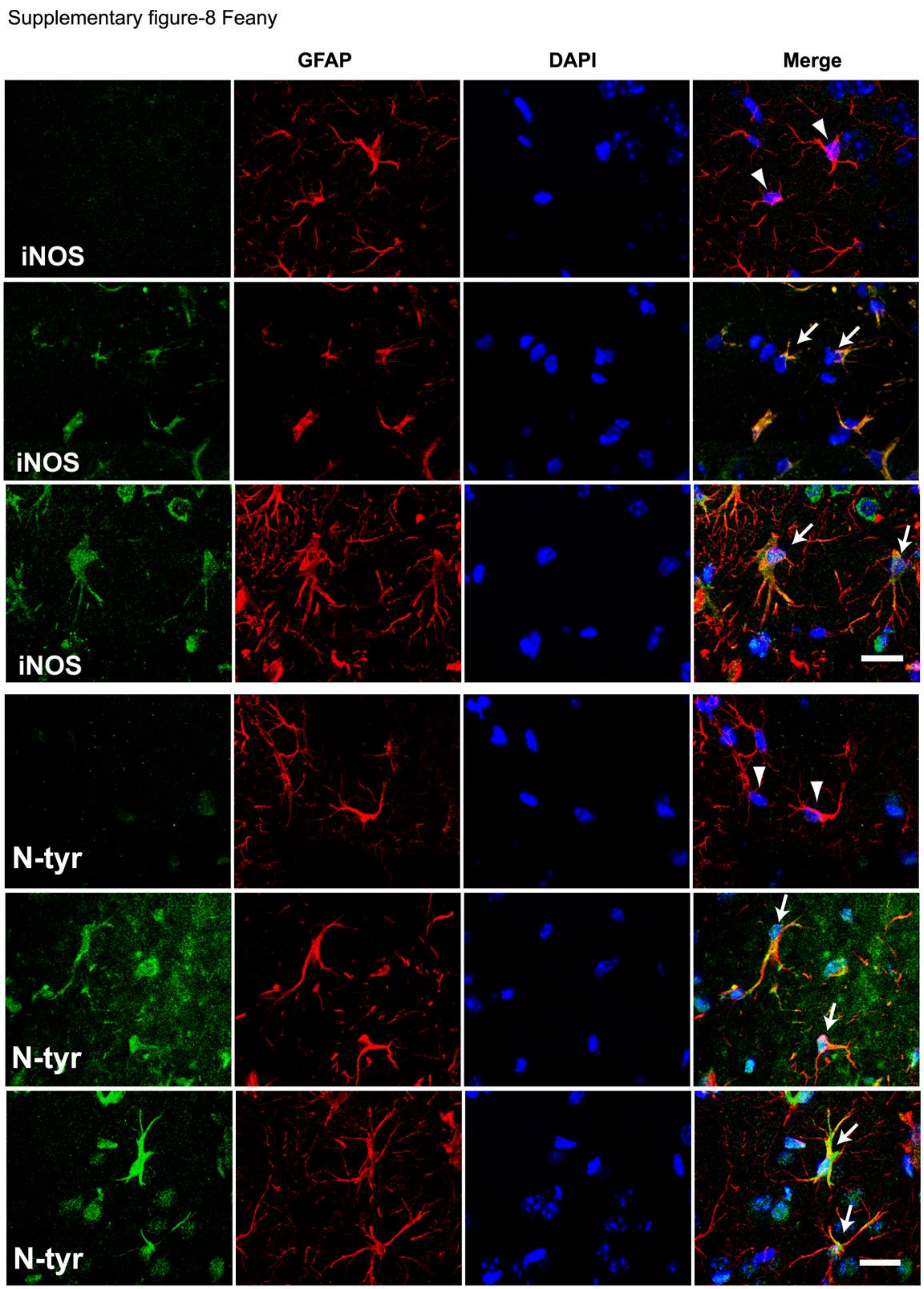

Supplementary Figure 8. Larger images of iNOS and N-tyr images in **Figure 4c**.

Supplementary figure-9 Feany

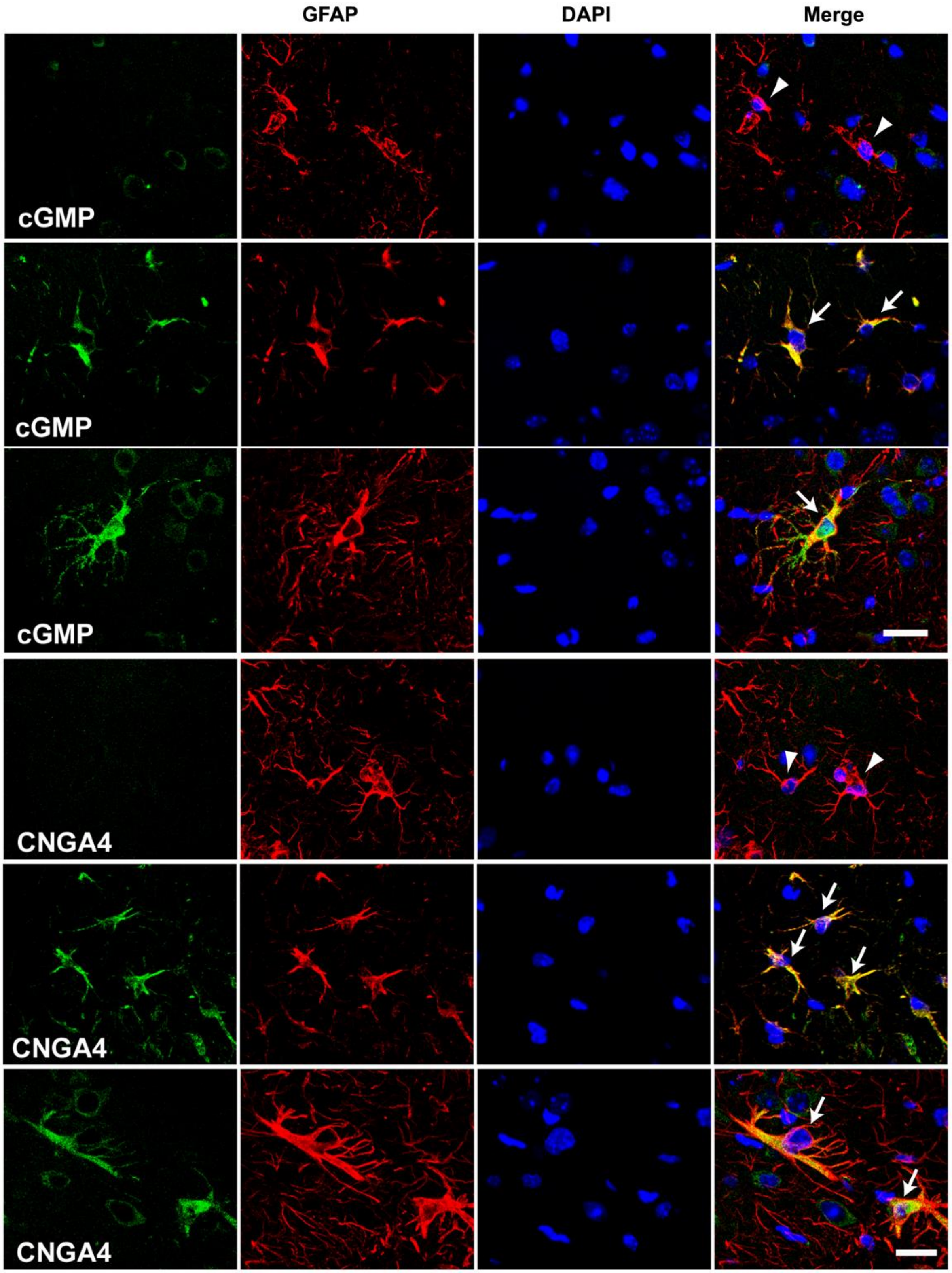

Supplementary Figure 9. Larger images of cGMP and CNGA images in **Figure 4c**.

Supplementary Table 1. List of antibodies, dilution factors and sources

Antibody	Western	Immunofluorescence	Source
actin	1:4,000		DSHB (clone JLA20)
β-galactosidase		1:500	Promega Z3781
cGMP		1:10	Sigma G4899
CNGA4		1:50	DSHB (clone 7B11)
Cleaved Dcp-1		1:100	Cell Signaling 9578
elav		1:10	DSHB (clone 9F8A9)
GFAP	1:1,000,000	1:10,000	Dako Z0334
GFP	1:500	1:50	UC Davis/NIH NeuroMab Facility (clone N86/8)
H2Av-pS137 (Dm)	1:20,000	1:500	Rockland 600-401-914
H2AX- pS139 (M)		1:50	Cell Signaling 2577
H2AX- pS139 (M)		1:1,000	Millipore 05-636 (clone JBW301)
Nitrotyrosine	1:100,000	1:500	Millipore AB5411
Nitrotyrosine	1:10,000	1:100	Upstate 05-233 (clone 1A6)
Nos (Dm)	1:5,000		P.O'Farrell[17]
iNOS (M)	1:1,000	1:500	BD Bioscience 610332
iNOS (M)		1:1	Santa Cruz 7271 (clone C-11)
iNOS (H)	1:400		BD Bioscience 610431 (clone 54/ iNOS)
p53 (Dm)	1:100	1:1	DSHB (clone H3)
p53 (M)		1:500	Santa Cruz 6243
p53 (H)		1:400	Dako M7001 (clone DO-7)
p53-pS15		1:50	Cell Signaling 9284
Repo		1:10	DSHB (clone 8D12)

Notes: Dm: *Drosophila melanogaster*; M: mouse; H: Human
